# Fractal Design Boosts Extrusion-Based 3D Printing of Bone-Mimicking Radial-Gradient Scaffolds

**DOI:** 10.34133/2021/9892689

**Published:** 2021-11-23

**Authors:** Huawei Qu, Zhenyu Han, Zhigang Chen, Lan Tang, Chongjian Gao, Kaizheng Liu, Haobo Pan, Hongya Fu, Changshun Ruan

**Affiliations:** ^1^School of Mechatronics Engineering, Harbin Institute of Technology, Harbin 150001, China; ^2^Research Center for Human Tissue and Organs Degeneration, Institute of Biomedicine and Biotechnology, Shenzhen Institute of Advanced Technology, Chinese Academy of Sciences, Shenzhen 518055, China; ^3^University of Chinese Academy of Sciences, Beijing 100049, China

## Abstract

Although extrusion-based three-dimensional (EB-3D) printing technique has been widely used in the complex fabrication of bone tissue-engineered scaffolds, a natural bone-like radial-gradient scaffold by this processing method is of huge challenge and still unmet. Inspired by a typical fractal structure of Koch snowflake, for the first time, a fractal-like porous scaffold with a controllable hierarchical gradient in the radial direction is presented via fractal design and then implemented by EB-3D printing. This radial-gradient structure successfully mimics the radially gradual decrease in porosity of natural bone from cancellous bone to cortical bone. First, we create a design-to-fabrication workflow with embedding the graded data on basis of fractal design into digital processing to instruct the extrusion process of fractal-like scaffolds. Further, by a combination of suitable extruded inks, a series of bone-mimicking scaffolds with a 3-iteration fractal-like structure are fabricated to demonstrate their superiority, including radial porosity, mechanical property, and permeability. This study showcases a robust strategy to overcome the limitations of conventional EB-3D printers for the design and fabrication of functionally graded scaffolds, showing great potential in bone tissue engineering.

## 1. Introduction

The critical segmental bone defect is one of the main problems in orthopedics, which seriously affects patient's health and quality of life [1]. Although autogenous bone grafting has long been the gold standard for treatment, the number of available bones limits its widespread clinical application [2]. In this case, bone tissue engineering was proposed in 1987 to obtain artificial bone substitutes for replacement and regeneration [3]. An ideal bone scaffold should have the characteristics of the interconnected structure, mechanical support, permeability, bioactivity, and so on [4]. Among these, an interconnected porous structure can effectively promote cell proliferation and in-growth, and transportation of nutrients and oxygen, and thus enhancing tissue regeneration [5–7]. It is challenging but very important to balance the relationship between the porous structure, the mechanical property, and permeability.

Natural bone tissue has been confirmed with at least 12 levels of fractal-like organizations from the nanoscale to the macroscale [8]. The adult human bone is commonly composed of two main zones with a porosity gradient, which gradually decreases from the inner cancellous bone to the outer cortical bone. Cancellous bone, also named trabecular or spongy bone, has a high porosity of 50-90% and a large number of fractal-like bubble structures; while cortical bone has a low porosity of 5-10% [9]. To mimic the gradient structures of natural bone, some computer-aided design (CAD) methods, such as voronoi tessellation [10], triply periodic minimal surfaces [11], and topology optimization [12], have been used to design bone scaffolds. The manufacturing technologies corresponding to these design methods are mainly selective laser melting, electron beam melting, and stereolithography [11–13]. However, these technologies are not biocompatible due to the high processing temperature or the toxicity of photosensitive materials.

As an environment-friendly technology with low cost, good scalability, and a diverse choice of materials, extrusion-based three-dimensional (EB-3D) printing has been increasingly attractive for scaffold fabrication [14, 15]. Furthermore, with a combination of cells and biomaterials as bioinks, this printing method provides an exciting opportunity to fabricate cell-laden tissue constructs [16–18]. Conventional approaches of EB-3D printing often possess a lay-down pattern of 0°/90° [19], and have an intrinsic shortcoming in preparing complex structures with gradients, especially radial gradients. Therefore, it is very challenging to design and fabricate functionally graded scaffolds that mimic natural bone structure based on EB-3D printing technology [20]. For the material gradient, the previous studies focused on building mixture platforms of EB-3D printing [21–24]. For the structural gradient, most previous investigations concerned the axial gradient, and the main strategy was to transform and upgrade conventional scaffolds with a lay-down pattern of 0°/90° by adjusting the filament diameter over different layers and the filament spacing within the same layer [20, 25–28]. However, there are few reports on the fabrication of radial-gradient structures. Although some studies reported that radial gradient scaffolds could be obtained by setting different fiber diameters and spacing in radial diverse zones of a target model, it is still not an easy task to produce a continuous, controllable, and gradient porous scaffold with high precision [9, 29].

Koch snowflake, a snowflake-shaped pattern, was discovered by Helge von Koch in 1904 [30]. This infinite-length curve is an ideal model for various structures in the real world, such as coastlines and bones [31]. In 1975, Benoit B. Mandelbrot coined the term “Fractal” to describe the shape in which detailed information existed at all scales in nature [32]. Simple curves or structures can repeat into self-similar 2D or 3D results through iterations of fractal mathematics. It has been a consensus that fractal mathematics is suitable for describing natural systems (i.e., lungs, blood vessels, and trees) [8, 31, 33, 34]. Fractal curves (i.e., Hilbert and Sierpinsky) have also been used to build infill space of 2D and 3D structures [16, 35].

**Figure 1:**
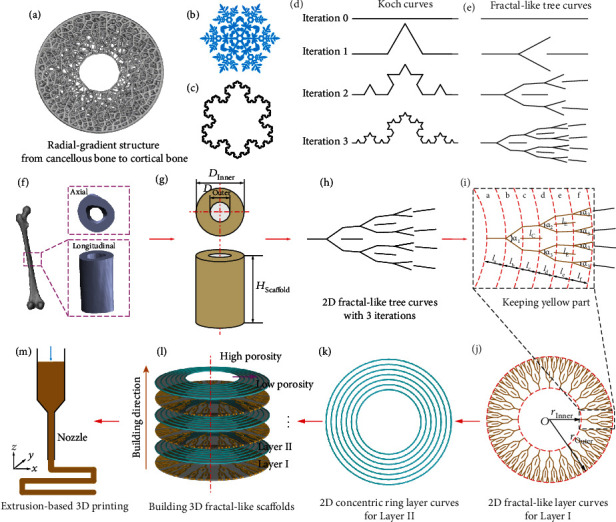
Schematic illustrations for designing fractal-like scaffolds. (a) Radial-gradient structure of a cross section of natural bone from cancellous bone to cortical bone. (b) Digital model of a natural snowflake. (c) Koch snowflake with 3 iterations. (d) Koch curves with 0, 1, 2, and 3 iterations, respectively (Figure S1, Supporting Information). (e) 2D fractal-like tree curves with 0, 1, 2, and 3 iterations, respectively (Figure S2, Supporting Information). (f) Digital model of adult femur based on micro-CT reconstruction. (g) Ideal hollow cylinder model obtained by circumscribing the ROI of the reconstructed model. (h) 2D fractal-like tree curves with 3 iterations. (i) Trimming the obtained 2D fractal-like tree curves. The yellow part was kept to building the porosity gradient in the radial direction. (j) 2D fractal-like layer curves obtained by a circular array of the trimmed curves. (k) Design of the 2D concentric ring layer curves for supporting the 2D fractal-like layer curves in the axial direction. (l) 3-iteration fractal-like 3D scaffolds obtained through a linear array of the 3D fractal-like layer (Layer I) and the 3D concentric ring layer (Layer II). (m) Fabrication G-code generation for controlling the printhead movement to deposit inks along the as-designed route.

Herein, inspired by the Koch snowflake, a novel bone-mimicking scaffold with different radial-gradient structures, recorded as a fractal-like scaffold, was proposed (Figure 1 and movie S1) and fabricated by EB-3D printing. The construct gradient of the fractal-like scaffold in the radial direction could be controlled by varying the filament diameters, the number of iterations, and the fractal parameters. A design-to-fabrication workflow was developed to build a bridge between the parametric design of the CAD models and the EB-3D printing of the fractal-like scaffold. This workflow provides a viable possibility for extending the application scope of traditional EB-3D printing techniques to fabricate complex structures. We further demonstrated that this strategy was capable of creating fractal-like scaffolds with controllable radial gradients in porosity, permeability, and mechanical property. Overall, the synergy of the fractal theory and design-to-fabrication workflow could shed light on more advanced porous scaffolds and functionally graded materials for bone tissue engineering.

## 2. Results

### 2.1. Design of Bone-Mimicking Radial-Gradient Scaffolds

Compared with previous reports on the construction of radial-gradient scaffolds by depositing different filament diameters and spacing in diverse radial zones [9, 29], as shown in Figure 1, the novel bone-mimicking radial-gradient scaffold is developed using fractal design based on EB-3D printing (movie S1), which is to imitate the radial-gradient structure of natural bone with gradually decreasing porosity from cancellous bone to cortical bone (Figure 1(a)). Natural snowflake (Figure 1(b)) and Koch snowflake (Figure 1(c)) exhibit self-similarity. A Koch snowflake, consisting of three Koch curves, is iterated from an equilateral triangle. We displayed four stages of a Koch curve (Figure 1(d), Figure S1, and movie S1) and designed 2D fractal-like tree curves with 0, 1, 2, and 3 iterations (Figure 1(e), Figure S2, and movie S1) using the 3D modeling software Rhino (also known as Rhinoceros or Rhino3D) integrated with the algorithmic 3D modeling tool Grasshopper. The 2D fractal-like tree curves with 0, 1, 2, and 3 iterations have 0, 1, 2, and 3 branches, respectively. We could tune the values of the branching angles (*α*, marked as fluorescent green in Figure S2) to regulate the distribution areas of the 2D fractal-like tree curves. In addition, odd-numbered line segments of the fractal-like tree curves are all set along the radial direction.

Taking the 3-iteration fractal-like 2D tree curves to construct the corresponding 3-iteration fractal-like 3D model as an example, the design flow diagrams are illustrated in Figures 1(f)–1(m). First, reconstruction of an adult femur model is developed based on micro-CT images from clinical diagnosis (Figure 1(f) and Figure S3), and a region of interest (ROI) is selected to determine the dimension of the hollow cylinder consisting of inner diameter (*D*_Inner_), outer diameter (*D*_Outer_), and height (*H*_Scaffold_) (Figure 1(g)). Second, to obtain the radial gradient, the as-designed 2D fractal-like tree curves (Figure 1(h)) are cut off the outermost line segments. The reserved and removed parts are marked in yellow and black, respectively (Figure 1(i)). Third, the trimmed 2D fractal-like tree curves are arranged in a circular array to constitute the 2D fractal-like layer curves (Figure 1(j)). Fourth, 2D concentric ring layer curves (Figure 1(k)) are developed to coincide with the branching point of the 2D fractal-like layer curves (Figure 1(j)) in the *z*-axis direction, supporting each other between different layer curves. Fifth, through using the command Pipe in the software Grasshopper, the 2D fractal-like layer curves and the 2D concentric ring layer curves are converted into the 3D fractal-like layer (Layer I, Figure 1(l)) and the 3D concentric ring layer (Layer II, Figure 1(l)), respectively. Sixth, the two base layers are linearly arranged along the axial direction with twice the filament diameter as the array spacing to build the bone-mimicking radial-gradient scaffold 3D model with the 3 iterations fractal-like structure (Figure 1(l)). Finally, we rewrite the fabrication codes (G-codes) to control the printhead movement of our EB-3D printer along with the designed pattern to deposit ink (Figure 1(m)).

**Figure 2:**
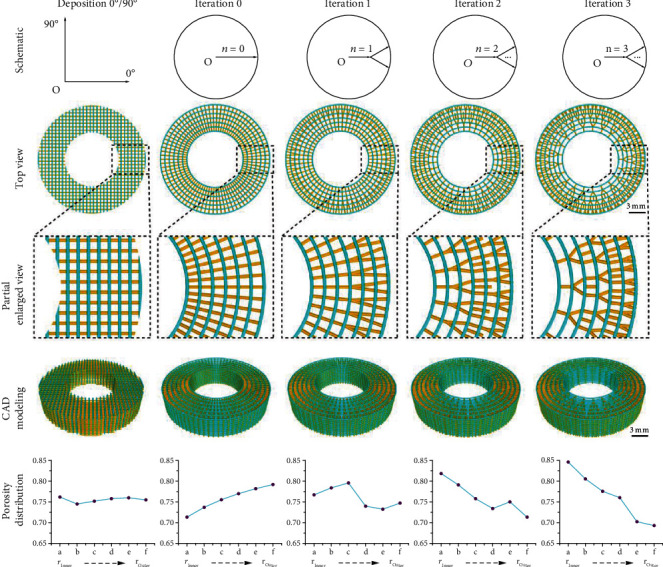
Schematic, top view, partially enlarged view, CAD model, and porosity distribution of the conventional and fractal-like scaffold models with different radially graded porosity.

The bone-mimicking radial-gradient scaffold models with different gradients can be obtained based on fractal design by adjusting the fractal parameters including filament diameters (*D*_Filament_), number of circular arrays (*NCA*), and branching angles (*α*). Five styles of porous scaffold models, including a conventional lay-down pattern of 0°/90° and fractal-like structures with 0, 1, 2, and 3 iterations, were obtained at the similar global porosity of 75.25 ± 0.14% and *D*_Filament_ of 200 *μ*m (Table S1).  Figure 2 shows the schematic, top view, partially enlarged view, CAD model, and local porosity distribution of these five scaffold models. All models were equally divided into six zones in the radial direction from the inner to the outer zone (Figure 1(i) and Figure S4). The porosity of each zone, known as the local porosity of the scaffold model, was processed with the Boolean operation via Bruker software CT-Analyser (CTAn). The porosity gradient of the conventional scaffold modes with 0°/90° deposition is constant at ~75% with small fluctuations; the 0-iteration and 3-iteration fractal-like structures are increasing and decreasing step by step, respectively; and the fractal-like scaffold modes with 1 and 2 iterations do not show a regular gradient distribution. Furthermore, it is worth noting that the 3-iteration fractal-like scaffold models have a gradient of gradually decreasing porosity from the inner to the outer zone, which is similar to natural bone.

### 2.2. Design-to-Fabrication Workflow

**Figure 3:**
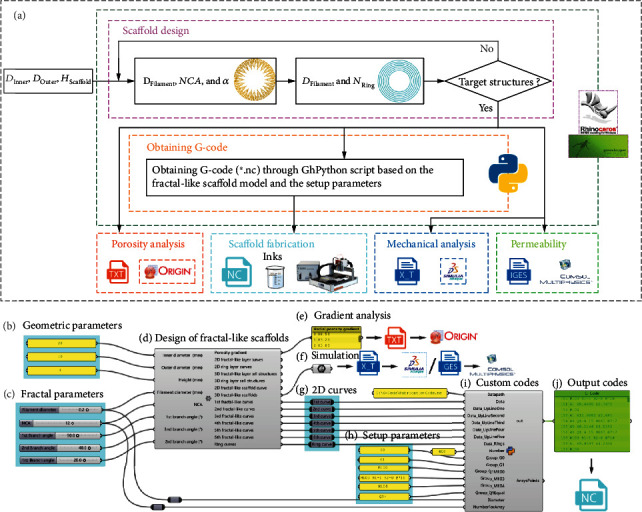
Customized design-to-fabrication workflow for the fractal-like scaffolds with 3 iterations. (a) Schematic diagram of the digital workflow. (b)-(i) Realization of digital workflow in visual programming. Grasshopper, as a visual programming plug-in of Rhinoceros software, is used to realize the carrier of the workflow of the fractal-like scaffolds. The customized components in this workflow include: (b) Geometric parameters, (c) Fractal parameters, (d) Design of fractal-like scaffolds, (e) Gradient analysis, (f) 3D model export for simulation, (g) 2D curves, (h) Setup parameters based on the printer's code rules, (i) Custom codes, and (j) Output G-codes.

A corresponding design-to-fabrication workflow is established based on the 2D curves of the parametric design and the printhead movement rules of our conventional EB-3D printer, which builds a bridge between designing a filament-stacked fractal-like model and fabricating a 3D printed sample, and also constructs the front and back analysis functions of porosity, mechanical properties, and permeability. (Figure 3). The novel bone-mimicking radial-gradient scaffold models with different geometrical dimensions and radial gradient porosity can be obtained by adjusting the geometric parameters (*D*_Inner_, *D*_Outer_, and *H*_Scaffold_) and the fractal parameters (*D*_Filament_, *NCA*, and *α*) (Figure 3(a)). Figure 3(a) schematically shows the flow chart of this workflow, and Figures 3(b)–3(j) illustrates its implementation circuit in software Grasshopper through the built-in battery packs. In particular, we customize and encapsulate a novel battery used to design the fractal-like scaffolds with 3 iterations. For this battery, part of the input parameters is geometric parameters (Figure 3(b)) obtained from the ROI at the femoral defect, which consists of *D*_Inner_, *D*_Outer_, and *H*_Scaffold_. The other part is fractal parameters (Figure 3(c)) used to control the 3D fractal-like layer of the 3D scaffolds, which consists of *D*_Filament_, *NCA*, and *α*. In addition, the parameter of the ring layer is set, where the value of *D*_Filament_ is the same as the fractal layer's, and the number of concentric rings (*N*_Ring_) is 7 (*N*_Ring_ =7). The output contains the gradient analysis (Figure 3(e)), 3D scaffold models for finite element analysis (FEA) and computational fluid dynamic (CFD) simulations (Figure 3(f)), and the 2D curves (Figure 3(g)) including the 2D fractal-like layer curves and the 2D ring layer curves. Moreover, GhPython Script, a built-in battery in the Python environment of the Grasshopper, is used to rewrite the fabrication codes (Figure 3(i)). Specifically, the GhPython Script is applied to convert the 2D curves (Figure 3(g)) into the fabrication codes NetCDF file (∗.nc) (Figure 3(j)) based on the code rules of the conventional EB-3D printer (Bioscaffolder 3.1, GeSiM, Germany) (Figure 3(h)). The 3-iterations fractal-like scaffolds are fabricated by the printer Bioscaffolder 3.1 via calling the customized fabrication codes (Figure 3(j) and Figure S5). This workflow allows us to modify the existing extrusion printer to meet the fabrication requirements of the designed model and provides an extension of simulation analysis.

### 2.3. 3D Printed Bone-Mimicking Radial-Gradient Scaffolds

**Figure 4:**
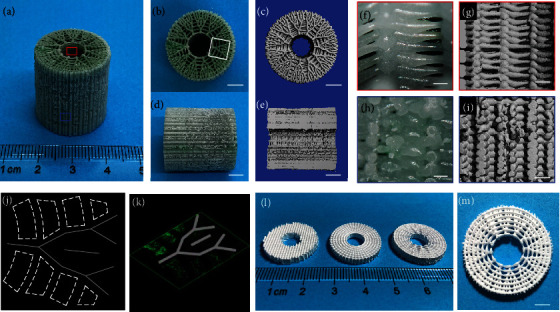
3D printed bone-mimicking radial-gradient scaffolds. (a)-(i) Photos of the 3D printed fractal-like *β*-TCP/PCL and dye/Alg sample with 3 iterations, which are axonometric drawings (a), axial drawings ((b) and (c)), longitudinal drawings ((d) and (e)), enlarged drawings of the inner scaffolds ((f) and (g)), and enlarged drawings of the outer scaffolds ((h) and (i)). The red and blue boxes represent the inner and outer structures of the 3-iteration fractal-like scaffolds, respectively. (j) and (k) Top and axial side views of live (green)/dead (red) CLSM images of the white box part (b) of the bio-printed fractal-like scaffolds. (l) and (m) Pictures of the 3D printed fractal-like PLGA sample. (a), (b), (d), (f), and (h) are optical images; (c), (e), (g), and (i) are CT images. Scale bars, 500 *μ*m ((f) and (h)); 1 mm ((g) and (i)); 5 mm ((b)-(e), and (m)).

Based on the design-to-fabrication workflow, we selected geometric parameters of human femoral size (*D*_Inner_ =8 mm, *D*_Outer_ =22 mm, and variable height according to demand) to construct bone-mimicking radial-gradient scaffolds. As shown in Figure 4, we verified the printability of the proposed critical-sized bone-mimicking scaffolds with different ink materials including *β*-TCP/PCL (*β*-tri-Calcium phosphate, *β*-TCP; Polycaprolactone, PCL) ink via a thermal-assisted EB-3D printing method, PLGA (poly(lactide-co-glycolide)) via a solvent-assisted low-temperature EB-3D printing strategy, and GelMA/Alg/hMSCs (gelatin methacryloyl, GelMA; pure alginate, Alg; human mesenchymal stem cells, hMSCs) bio-ink via a mild bioprinting method. The EB-3D printing process of the PLGA samples with three structures is displayed in movies S2–S4. To vividly demonstrate the bioprinting process, we use a dye/Alg ink instead of the GelMA/Alg/hMSCs bioink (fabrication process see movie S5). Figures 4(a)–4(i) illustrate the critical-sized bone-mimicking models with the 3-iteration fractal-like structure. As reported in the previous research, a cortical-like outer shell scaffold has been proven to promote bone regeneration in the central area and prevent fibroblasts from growing into the scaffold [36]. In this work, the inner and outer shell of the 3-iteration fractal-like scaffolds are somewhat similar to cancellous bone and cortical bone, respectively. In detail, the former has good porosity (Figures 4(f) and 4(g), and Figure S6), while the latter has almost no porosity (Figures 4(h) and 4(i), and Figure S6). The bio-printing of the 3-iteration fractal-like scaffolds are composed of fractal layers, ring layers, and bioink layers, wherein the space of the bioink layers is the gap between the concentric rings in each ring layer. The fractal and ring layers are given as *β*-TCP/PCL, and the bioink layers are given as GelMA/Alg/hMSCs. The confocal laser scanning microscope (CLSM) pictures (Figures 4(j) and 4(k)) of the 3D bio-printed fractal-like scaffolds (design parameters shown in Table S2) show that the number of hMSCs sequentially decreases from the inner to the outer zone in the radial direction. We have qualitatively and quantitatively analyzed the bioprinted scaffolds with a conventional lay-down pattern of 0°/90° and fractal-like structures with 0 iterations and 3 iterations, as shown in Figure S7 (Supporting Information). The live/dead staining demonstrated that ~78% of the cells survived on day 1. The cell spreading on the three structure scaffolds can be obviously observed on day 5, which proves the feasibility of bioprinting in the bone-mimicking radial-gradient structure. In addition, we demonstrate the feasibility of the proposed bone-mimicking radial-gradient scaffolds for solvent ink printing (Figures 4(l) and 4(m)).

### 2.4. Pore Characteristics, Mechanical Properties, and Permeability of the Bone-Mimicking Radial-Gradient Scaffolds

**Figure 5:**
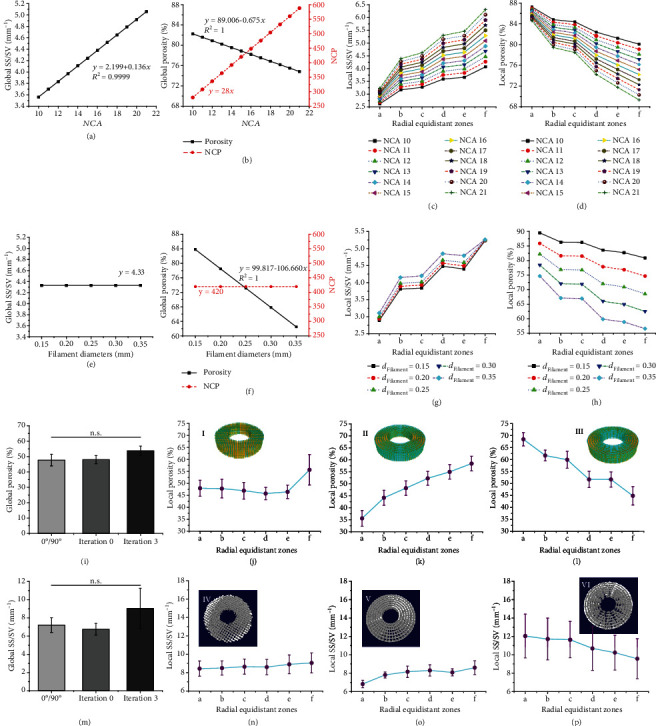
Parametric characterization of the 3D printed PLGA scaffolds with functionally graded constructs. (a)-(h) Porosity and SS/SV of the global and local fractal-like scaffolds with 3 iterations controlled by *NCA* and *D*_Filament_. (a)-(d) Effects of the *NCA* on the porosity and SS/SV of the global and local fractal-like scaffolds when the same of nozzle diameter (200 *μ*m) and three-level branching angles (*α* =33°, 25°, and 12°, respectively). (e)-(h) Effects of the filament (nozzle) diameters on the porosity and SS/SV of the global and local fractal-like scaffolds at the same iteration number (iteration =3), *NCA* =15, and three-level branching angles (*α* =43°, 34°, and 12°, respectively). (i)-(p) The porosity and SS/SV of the global and local scaffolds with three different graded constructs at the same porosity of ~76%. (i) The porosity of the global scaffolds. (j)-(l) The porosity of the local scaffolds with 0°/90° deposition (j) and fractal-like structures with 0 iterations (k) and 3 iterations (l). (m) The SS/SV of the global scaffolds. (n)-(p) The SS/SV of the local scaffolds with 0°/90° deposition (n) and fractal-like structures with 0 iterations (o) and 3 iterations (p). The CAD models (I-III) and 3D printed samples (IV-VI) of the scaffolds with three different gradients are represented. The designed and CT-reconstructed models are equally divided into six zones (from zone a to zone f) in the radial direction from the inner to the outer zone. Four samples (n = 4) of each type of 3D printed scaffolds are tested.

Figure 5 shows the influence of the fractal parameters (Figures 5(a)–5(h)) and the number of iterations (Figures 5(i)–5(p)) on the pore characteristics of the bone-mimicking radial-gradient scaffolds. To evaluate how the fractal parameters affect the scaffold porosity and the scaffold surface to scaffold volume ratio (SS/SV) at global and local scales, the 3-iteration fractal-like scaffold models with different pore structures are designed and obtained by changing the fractal parameters including the *D*_Filament_, *NCA*, and *α*, as shown in Figures 5(a)–5(h). As only the value of the *NCA* increases at the same nozzle diameter (*D*_Filament_ =200 *μ*m) and three-level branching angles (*α*_1_ =33°, *α*_2_ = 25°, and *α*_3_ = 12°, respectively), it is noticed that the global and local porosity decrease, but the global SS/TV, the gradient in the local porosity and the local SS/SV, and the number of contact points (NCP) increases (Figures 5(a)–5(d)). As only the value of the *D*_Filament_ increases at the same number of iterations (iteration =3), *NCA* values (*NCA* =15), and three-level branching angles (*α*_1_ =43°, *α*_2_ =34°, and *α*_3_ = 12°, respectively), it is observed that the global and local porosity decrease, and the global SS/SV and the NCP remain unchanged, but the local SS/SV increase (Figures 5(e)–5(h)). Meanwhile, NCP is explored by adjusting the above parameters (Figures 5(b)–5(f)), and we observe a linear relationship between NCP and *NCA*.

To assess the number of iterations, three groups with different radial gradients (design parameters shown in Table S3), including a lay-down pattern of 0°/90°, 0-iterations and 3-iteration fractal-like structures, were designed at the same global porosity of 75.87 ± 0.15% and the same *D*_Filament_ of 300 *μ*m (designed models shown in Figures 5(i)-(III)). The U.S. Food and Drug Administration (FDA) approved PLGA was selected as a raw material for EB-3D printing [37]. The PLGA samples were obtained by our commercial EB-3D printer based on the developed design-to-fabrication workflow (reconstruction models of 3D-printed PLGA samples based on micro-CT, as shown in Figure 5(IV)-4(VI)). The EB-3D printing process of the PLGA samples is displayed in movies S2–S4. The deposition sequence of the fractal layer of the 3-iteration fractal-like scaffolds is shown in Figure S8 and movie S4. The porosity and the SS/SV of the global and local 3D printed PLGA scaffolds are summarized (Figures 5(i)–5(p)). The porosity and the SS/SV of the global scaffolds have no mathematically significant difference among these three graded structures (Figures 5(i) and 5(m)). As one control group, the local porosity and the local SS/SV of the 3D printed conventional scaffolds do not significant fluctuation, except for the unusually high rise in zone f (Figure 5(j)). The 0-iteration fractal-like group, another control group, has a local porosity that gradually increases from the inner to the outer zone (Figure 5(k)), which is a reverse radially gradient porosity from cancellous bone to cortical bone. The 3-iteration fractal-like group tends to gradually decrease in the radial direction, which is similar to the porosity gradient of natural bone (Figure 5(l)). The local SS/SV of the three groups has similar changes following the fluctuation of porosity, but the change gradient is not as obvious as their porosity (Figures 5(n)–5(p)). A one-way ANOVA analysis was performed on the local porosity and the local SS/SV of the six zones (Figure S4) of each reconstructed model, and only the local porosity of the fractal-like scaffolds with 0 and 3 iterations had a significant difference (Figure S9).

**Figure 6:**
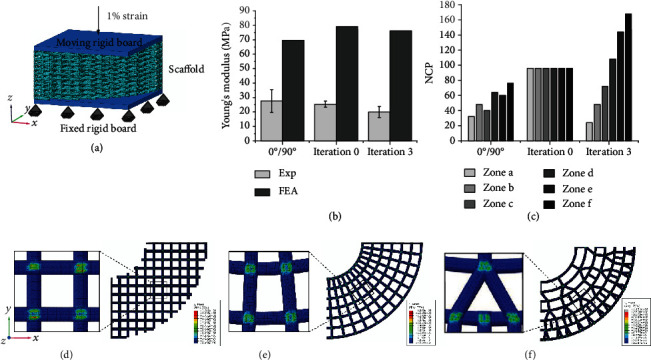
Mechanical properties of the PLGA scaffolds with a lay-down pattern of 0°/90°, 0 iterations, and 3 iterations. (a) Schematic diagram of uniaxial compression experiment. (b) Experiment and FEA simulation of uniaxial compression of the porous scaffolds and relationship between the compressive modulus and the NCP. (c) The local NCP of the porous scaffolds with three different graded constructs at the same porosity. (d)-(f) Von-Mises stress distribution in FEA models with three porous structures, which are the lay-down pattern of 0°/90°, 0-iteration fractal-like structure, and 3-iteration fractal-like structure from left to right, respectively.

For the mechanical properties of the three kinds of porous scaffolds, a quarter of each scaffold model obtained by the parametric design is converted into a mesh model for the FEA simulation (Figure S10). The boundary conditions of the FEA modeling are set to simulate the uniaxial compression process of the 3D printed scaffolds (Figure 6(a)). Although the simulated compressive modulus is abnormally higher than the experimental modulus, the mechanical properties of the two results are similar, and the three types of scaffolds have little fluctuations and no significant difference (Figure 6(b) and Figure S11). Figure 6(c) shows the local NCP of the porous scaffolds with three different graded constructs at the similar mechanical properties. In addition, Von-Mises stress distributions of the FEA models with 0°/90° deposition, 0 iterations, and 3 iterations are shown in Figures 6(d)–5(f) and Figure S12, and their color mutation position is located at the contact points between fractal layers and ring layers. Although the NCP and the compressive modulus have no obvious similar change trend, the NCP of the bone-mimicking radial-gradient with a 3-iteration fractal-like structure gradually increases along the radial direction, which meets the requirements for mechanical support performance of natural bone from cancellous bone to cortical bone.

**Figure 7:**
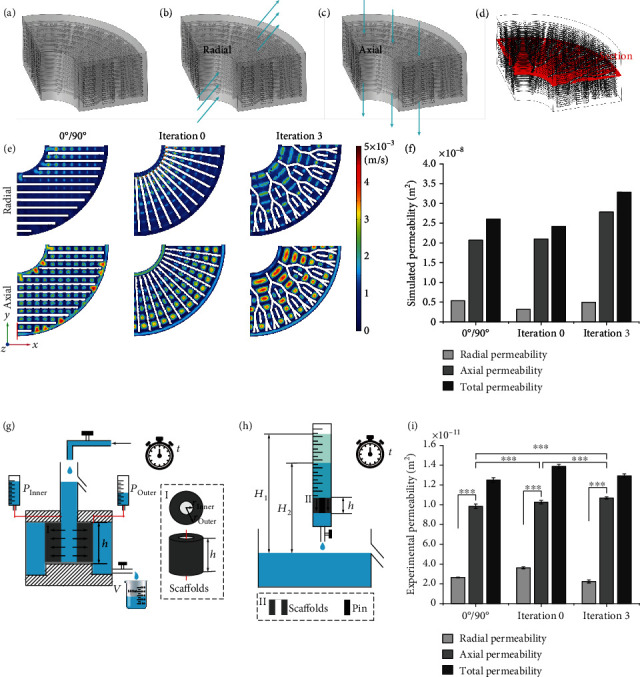
Permeability of the 3D printed *β*-TCP/PCL scaffolds with a lay-down pattern of 0°/90°, 0 iterations, and 3 iterations. (a) Taking a quarter of the fluid computation domain obtained by the Boolean subtraction operation as the domain of interest due to the symmetry of the model. (b)-(c) Schematic diagram of radial (b) and axial (c) permeability testing. (d) Selecting a cross-section of the domain of interest to show the results of fluid flow simulation. (e) The flow rate of CFD simulations of the three porous structures. (f) Quantification of permeability simulation of the CAD scaffold models. (g)-(h) Schematic diagrams of the radial (g) and axial (h) permeability setups used to test the 3D printed scaffolds. Insets (I) and (II) are an enlarged view of the position of the scaffolds in the schematic of axial permeability tests and a top view and a longitudinal view of the scaffolds in the schematic of radial permeability tests, respectively. To prevent the liquid from passing through the central blank area of the 3D printed *β*-TCP/PCL scaffolds, a solid pin is placed in this area. (i) Quantification of permeability tests of the 3D printed scaffolds.

For the fluid flow of the three kinds of porous scaffolds, a fluid computation domain is obtained by Boolean subtraction, symmetrical processing, and meshing (Figure S13). A quarter of this domain is imported into software COMSOL Multiphysics 5.4 as IGES (Initial Graphics Exchange Specification) files from software Rhino (Figure 7(a)). Schematic diagrams of the radial permeability and the axial permeability are shown in Figures 7(b) and 7(c), respectively. Figure 7(e) shows the CFD simulation results of the radial and axial fluid flow velocities of the different domains in the top view of the section in Figure 7(d) (top view of the different domains shown in Figure S14). The radial and axial permeability of the porous scaffolds can be developed according to Darcy's law [9], and the total permeability is defined as the sum of the radial and axial permeability. The axial permeability of the CFD simulations of all models (2.069 × 10^−12^ m^2^ for the lay-down pattern of 0°/90° structure, 2.103 × 10^−12^ m^2^ for the 0-iteration fractal-like structure, and 3.246 × 10^−12^ m^2^ for the 3-iteration fractal-like structure), is better than their radial permeability (Figures 7(e) and 7(f)). The axial permeability of the bone-mimicking scaffold with the 3 iterations fractal-like structure has an excellent fluid flow gradient from the inner to the outer (Figure 7(e)). The schematic diagrams of the radial and axial permeability systems are shown in Figures 7(g) and 7(h), respectively. Assembly drawings and pictures of the permeability setups can be found in Figure S15. The axial permeability of the experimental results (Figure 7(i); experimental process shown in Supporting Information) of all models is better than the radial permeability in line with the trend of simulated permeability, and it is a significant difference between the axial permeability and the radial permeability of each group of the 3D printed *β*-TCP/PCL scaffolds (Figure 7(i)). In addition, the experimental axial permeability of the three style scaffolds all have significant differences compared with the experimental axial permeability, and the 3-iteration fractal-like structure has the best axial permeability ((106.911 ± 1.008) × 10^−12^ m^2^), as illustrated in Figure 7(i).

## 3. Discussion

We have developed a novel bone-mimicking radial-gradient scaffold based on a customized design-to-fabrication workflow, enabling us to obtain controllable radial-gradient structures by EB-3D printing, which solves the thorny problem of traditional EB-3D printing in preparing gradient structures. (Table S4, Supporting Information). The parameterization of fractal design provides unlimited structural possibilities for porous scaffolds. Our proposed work is capable of designing the bone-mimicking radial-gradient scaffolds with any number of iterations, but due to the manufacturing accuracy of EB-3D printer, we only show the bone-mimicking scaffolds with 0 to 3 iterations in this article, and 3-iteration strategy can already achieve the bone-mimicking radial-gradient scaffolds with a gradual decrease in porosity along the radial direction. We highlight our novel bone-mimicking scaffolds with the 3-iteration fractal-like structure to demonstrate the superiority of the radial-gradient pattern, including radial porosity, mechanical property, and permeability. The mechanical property of the bone scaffold provides appropriate support for the defect site, stimulates bone regeneration and repair, and provides interconnected pores to support the growth of new bone. The different radial zones of the 3-iteration fractal-like group have different local porosity and correspond to diverse potential functions: (1) The innermost cavity zone is used to connect the bone marrow cavity of the healthy femur; (2) The porous zone in the middle simulates cancellous bone and provides enough space for nutrient delivery, cell survival, and growth; (3) The outermost dense zone mimics cortical bone, offers mechanical support for tissue repair and regeneration, and prevents fibrous tissue from growing in and hindering tissue regeneration. For permeability, the previous reports mainly focus on the axial permeability of cylindrical or cube-shaped models [28]. In this study, the radial and axial permeability setups were newly developed for testing hollow cylindrical samples and were manufactured using acrylic glass plates. Compared with traditional structural scaffolds, our proposed bionic radial-gradient scaffold suitable for the direct ink writing printing method has obvious radial-gradient permeability [38]. This new design has the potential to serve as a robust strategy for overcoming the limitations of conventional EB-3D printers in the design and fabrication of functionally graded scaffolds, showing great potential in bone substitution or regeneration.

For the human femur-sized fractal-like scaffold, we have proved the outstanding performance of radial porosity, mechanical properties, permeability, and bioprintability, but there is a lack of in-depth cell experiments and critical-sized bone defects animal experiments. Compared with the significant stimulation of different biomaterials on cellular functions [18, 39, 40], the impact of varying structures is slightly inferior. *In vitro* cell experiments may not be enough to demonstrate the properties of our proposed structure in bone repair and regeneration. Unfortunately, directly performing large-size repair operation of the patient's femur with the human-sized bone-mimicking scaffolds also faces great ethical and practical challenges. In the future, we plan to conduct animal experiments to comprehensively evaluate the performance of this structure in bone tissue engineering, particularly large-size femoral defects with bone marrow cavity [1, 41]. In addition, some studies have proved that large pores (diameter ≥ 100-300 *μ*m) are obvious for the growth of blood vessels in the porous scaffolds [4]. We believe that the gradient pores of this bone-mimicking scaffold will promote blood vessel growth, showing its advantages in bone regeneration.

Although bone-mimicking scaffold models that fit the size of rabbits, goats, pigs, and other small animals can be easily obtained by the parametric design method proposed in this work, their fabrication is still a thorny problem due to the unsatisfying precision of current EB-3D printers. On the one hand, *D*_Filament_ limits the increase in the iteration numbers of a fractal-like scaffold under a certain geometric size. On the other hand, to obtain a high gradient of the fractal-like scaffold by increasing the *NCA* of the fractal-like tree curves, the fractal layer may not be able to support the innermost circle of the ring layer due to its large span, leading to collapse and loss of pores. High-precision printing technologies, such as melt electrowetting [42], and microfluidic systems [43], can be used to improve the processing accuracy and build micro-scale fractal-like scaffolds. To solve the large-span problem, future studies should involve sacrificial materials such as Pluronic F127 to support the fabrication of the fractal-like scaffolds or develop high-strength materials as inks [44–46].

In addition, replacing the initial 2D tree-like fractal curves used in this work with other patterns and/or changing the fractal iteration rules could produce unexpected effects and expand other gradient functions of the fractal-like scaffolds. For instance, the improved fractal-like scaffolds could be extended to other tissues such as blood vessels and trachea [31] via adjusting the fractal curves, fractal parameters, and the number of iterations and using coaxial printheads instead of traditional nozzles [47]. This workflow could be a promising technological improvement, not only because the fractal and parametric design provide infinite possibilities for the creation of CAD models, but also because the rewritten fabrication codes can redefine and redevelop the conventional EB-3D printers that lack gradient data. Given the great flexibility of this work, it can be extended and applied to design other porous scaffolds or functionally graded materials/structures and solve the dilemma where conventional EB-3D printers cannot precisely fabricate complex structures.

## Data Availability

All data needed to evaluate the conclusions in the paper are present in the paper and/or the Supplementary Materials. Additional data, including raw videos, may be requested from the authors.

## Supplementary Materials

Supplementary Materials Figure S1. Four iteration stages of the Koch snowflake (0, 1, 2, and 3 iterations). Figure S2. Four iteration stages of 2D fractal-like curves with 0, 1, 2, and 3 iterations. Figure S3. CT data of a 58-year-old Chinese woman femur. Figure S4. Definition of the local zones of the porous scaffolds in the radial direction for evaluating the local porosity and the local SS/SV. Figure S5. Establishing the software implementations of the design-to-fabrication workflow from the designed CAD models to the EB-3D printed samples. Figure S6. Axonometric and enlarged views of the 3D bio-printed fractal-like scaffolds with 3 iterations. Figure S7. Qualitative and quantitative analysis of live/dead staining for the bio-printed porous scaffolds on days 1, 3, and 5. Figure S8. Deposition sequence of the 2D fractal curves of the fractal-like scaffolds. Figure S9. Quantification of local porosity and local SS/SV of porous scaffolds in the radial direction. Figure S10. Main steps for converting CAD models into finite element meshes of the uniaxial compression. Figure S11. Uniaxial compression tests of the porous scaffolds. Figure S12. FEA simulation of uniaxial compression. Figure S13. Main steps for converting CAD models into fluid computation domains for the radial and axial permeability measurements. Figure S14. Top view of CFD simulation. Figure S15. Schematic diagrams, assembly drawings, and main pictures of the axial and radial permeability setups. Table S1. Design parameters of five types of scaffold models. Table S2. Design parameters of three types of scaffold models with different gradients. Table S3. Design parameters of 3D bio-printed of the 3-iterations fractal-like scaffolds. Table S4. Comparison of various design methods for bone tissue engineering scaffolds with porous graded structures. Movie S1. Design of bone-mimicking scaffolds based on fractal design. Movie S2. EB-3D printing of conventional PLGA scaffolds with a lay-down pattern of 0°/90°. Movie S3. EB-3D printing of fractal-like PLGA scaffolds with 0 iterations. Movie S4. EB-3D printing of fractal-like PLGA scaffolds with 3 iterations. Movie S5. EB-3D bio-printing of fractal-like *β*-TCP/PCL and dye/Alg scaffolds with 3 iterations.
