# Supplementary material for: Fractal Design Boosts Extrusion-Based 3D Printing of Bone-Mimicking Radial-Gradient Scaffolds
Source: Research (Wash D C). 2021 Nov 23;2021:9892689. doi: 10.34133/2021/9892689 (PMC8637129; doi:10.34133/2021/9892689)
Supplement: Supplementary Materials — Figure S1. Four iteration stages of the Koch snowflake (0, 1, 2, and 3 iterations). Figure S2. Four iteration stages of 2D fractal-like curves with 0, 1, 2, and 3 iterations. Figure S3. CT data of a 58-year-old Chinese woman femur. Figure S4. Definition of the local zones of the porous scaffolds in the radial direction for evaluating the local porosity and the local SS/SV. Figure S5. Establishing the software implementations of the design-to-fabrication workflow from the designed CAD models to the EB-3D printed samples. Figure S6. Axonometric and enlarged views of the 3D bio-printed fractal-like scaffolds with 3 iterations. Figure S7. Qualitative and quantitative analysis of live/dead staining for the bio-printed porous scaffolds on days 1, 3, and 5. Figure S8. Deposition sequence of the 2D fractal curves of the fractal-like scaffolds. Figure S9. Quantification of local porosity and local SS/SV of porous scaffolds in the radial direction. Figure S10. Main steps for converting CAD models into finite element meshes of the uniaxial compression. Figure S11. Uniaxial compression tests of the porous scaffolds. Figure S12. FEA simulation of uniaxial compression. Figure S13. Main steps for converting CAD models into fluid computation domains for the radial and axial permeability measurements. Figure S14. Top view of CFD simulation. Figure S15. Schematic diagrams, assembly drawings, and main pictures of the axial and radial permeability setups. Table S1. Design parameters of five types of scaffold models. Table S2. Design parameters of three types of scaffold models with different gradients. Table S3. Design parameters of 3D bio-printed of the 3-iterations fractal-like scaffolds. Table S4. Comparison of various design methods for bone tissue engineering scaffolds with porous graded structures. Movie S1. Design of bone-mimicking scaffolds based on fractal design. Movie S2. EB-3D printing of conventional PLGA scaffolds with a lay-down pattern of 0°/90°. Movie S3. EB-3D printin [file 9892689.f1.zip › Qu_SupplementalMaterial.docx]

Fractal Design Boosts Extrusion-Based 3D Printing of Bone-Mimicking Radial-Gradient Scaffolds

Huawei Qu^1,2^, Zhenyu Han^1^, Zhigang Chen^2^, Lan Tang^2,3^, Chongjian Gao^2^, Kaizheng Liu^2^, Haobo Pan^2,3*^, Hongya Fu^1*^, Changshun Ruan^2,3*^

^1^School of Mechatronics Engineering, Harbin Institute of Technology, Harbin 150001, China

^2^Research Center for Human Tissue and Organs Degeneration, Institute of Biomedicine and Biotechnology, Shenzhen Institutes of Advanced Technology, Chinese Academy of Sciences, Shenzhen 518055, China

^3^University of Chinese Academy of Sciences, Beijing 100049, China

Correspondence should be addressed to Hongya Fu; hongyafu@hit.edu.cn, and Haobo Pan; hb.pan@siat.ac.cn and Changshun Ruan; cs.ruan@siat.ac.cn

**Supplementary Materials**

**Materials and Methods**

**Figure S1:** Four iteration stages of the Koch snowflake (0, 1, 2, and 3 iterations).

**Figure S2:** Four iteration stages of 2D fractal-like curves with 0, 1, 2, and 3 iterations.

**Figure S3:** CT data a 58-year-old Chinese woman femur.

**Figure S4:** Definition of the local zones of the porous scaffolds in the radial direction for evaluating the local porosity and the local SS/SV.

**Figure S5:** Establishing the software implementations of the design-to-fabrication workflow from the designed CAD models to the EB-3D printed samples.

**Figure S6:** Axonometric and enlarged views of the 3D bio-printed fractal-like scaffolds with 3 iterations.

**Figure S7:** Qualitative and quantitative analysis of live/dead staining for the bio-printed porous scaffolds on days 1, 3, and 5.

**Figure S8:** Deposition sequence of the 2D fractal curves of the fractal-like scaffolds.

**Figure S9:** Quantification of local porosity and local SS/SV of porous scaffolds in the radial direction.

**Figure S10:** Main steps for converting CAD models into finite element meshes of the uniaxial compression.

**Figure S11:** Uniaxial compression tests of the porous scaffolds.

**Figure S12:** FEA simulation of uniaxial compression.

**Figure S13:** Main steps for converting CAD models into fluid computation domains for the radial and axial permeability measurements.

**Figure S14:** Top view of CFD simulation.

**Figure S15:** Schematic diagrams, assembly drawings, and main pictures of the axial and radial permeability setups.

**Table S1.** Design parameters of five types of scaffold models.

**Table S2.** Design parameters of three types of scaffold models with different gradients.

**Table S3.** Design parameters of 3D bio-printed of the 3-iterations fractal-like scaffolds.

**Table S4.** Comparison of various design methods for bone tissue engineering scaffolds with porous graded structures.

**Movie S1.** Design of bone-mimicking scaffolds based on fractal design.

**Movie S2.** EB-3D printing of conventional PLGA scaffolds with a lay-down pattern of 0°/90°.

**Movie S3.** EB-3D printing of fractal-like PLGA scaffolds with 0 iterations.

**Movie S4.** EB-3D printing of fractal-like PLGA scaffolds with 3 iterations.

**Movie S5.** EB-3D bio-printing of fractal-like β-TCP/PCL and dye/Alg scaffolds with 3 iterations.

**Materials and Methods**

**Ink preparation**

For the PLGA ink, PLGA (Daigang Biomaterials Co., Ltd., Jinan, Shandong, China) with a molecular weight of 100,000 and a 75:25 ratio of lactic acid to glycolic acid, and 1,4-dioxane (Aladdin Reagent Co., Ltd., Shanghai, China) were mixed in a weight-volume ratio of 40% (w/v) to prepare inks for direct ink writing (DIW) 3D printing. The mixture was magnetically stirred at room temperature (25℃) for 3 hours. The PLGA ink was used immediately after finishing mixing. For the β-TCP/PCL ink, β-TCP (Sigma-Aldrich, USA) and PCL (Aldrich, USA) with a molecular weight of 14,000 were prepared at a weight ratio of 25% (w/w). They were placed in a heating box at 75℃ for 1 hour and then stirred with a stick, repeating this process 3 times. The solution obtained in the previous step was pre-extruded by the DIW 3D printer using a 500 μm diameter nozzle heated to 72°C. For the GelMA/Alg/hMSCs bioink, it was composed of GelMA, and 2-hydroxy-2-methyl-1-phenyl-1-propanone (IRGACURE 1173, 98%, Sigma-Aldrich, St. Louis, USA) with a weight-volume ratio of 15%, 0.25% (w/v) to deionized water respectively. The synthesis method of GelMA used in this work was described in detail in the previous literature [[1](#_ENREF_1)]. The materials obtained from the above menu were stirred at room temperature (25℃) for 1 hour. The mixture and human mesenchymal stem cells (hMSCs, HUXMA-01001, Cyagen, China) were mixed uniformly at a ratio of 5×10^5^ cells/ml. The GelMA/Alg/hMSCs bioink was used immediately after finishing mixing. For the dye/Alg ink, to demonstrate the bio-printing process of the 3-iterations fractal-like scaffolds, we used pure Alg with green dye (Food coloring compound colorant, DAB Corporation, Japan) as a substitute for bioink layer. The grass green dye was mixed at 0.1% (v/v) into double distilled water at room temperature (25℃). Pure Alg (low viscosity, Sigma-Aldrich, USA) was added to the solution with a weight ratio of 45% (w/w). The mixture was manually stirred and mixed for 30 minutes.

**Scaffold design**

Porous scaffold models, including 0°/90° deposition and fractal-like structures with 0 to 3 iterations, were parametrically designed by the algorithmic modeling tool Grasshopper (htttp://www.grasshopper3d.com/) in the Rhinoceros 3D (Robert McNeel & Associates, v.6.0 SR21) at the same porosity (Figure 2). The design parameters of five types of scaffold models were summarized (see Table S1, Supporting Information). To control the radial gradient of the fractal-like scaffold models, several strategies were provided. The first was to change the *NCA* of the fractal curves of the fractal layer of fractal-like scaffold models (Figure 5(d)). The second was to adjust the number of iterations, as it increases, there was a significant gradient increase (Figure 5(j)-5(l)). The designed models could be exported in interesting formats, such as *.x_t and *.iges files, according to the needs of FEA modeling and CFD simulations.

**G-codes generation**

The function of the G-codes was to control the printhead to sequentially squeeze inks along the designed pattern to fabricate the samples. The conventional fabrication G-codes were usually obtained by automatically slicing 3D models based on the instruction laws of DIW 3D printers. In our developed design-to-fabrication workflow (Figure 3), the 2D concentric ring layer curves and the 2D fractal layer curves were used as the filling curve (Figure 1 and Figure 3(g)) with the instruction laws of our existing printer (Bioscaffolder 3.1, GeSiM, Germany) to customize the fabrication G-codes. The 3D fractal and layers were printed on the first and second layers respectively, which was repeated from the third layer until the required height of the scaffold. The deposition sequence of the 2D fractal curve with 3 iterations was defined (Figure S4, Supporting Information). In addition, before the printhead starts drawing each line segment, the air pressure of the extruder was turned 0.2 seconds in advance. After drawing each line segment, the air pressure of the extruder was turned off and the nozzle continues to move 2 mm in the current direction to break the continuous filaments. The important commands used in our 3D printer Bioscaffolder 3.1 are defined as follows: M103 is to open the air pressure; M103 R2=200 means that the delay of opening the air pressure is 200 milliseconds; M104 is to close the air pressure; R110 = 450 represents the printhead extrusion pressure is 500 kPa; G0 is a fast-moving and its speed is the system default; G1 is a slow-moving and its value can be customized; F540 means the printhead moving speed is 540 mm/min; and X13 Y14 is to move the printhead to the point where the X coordinate is 13 mm and the Y coordinate is 14 mm.

**Scaffold fabrication**

A commercialized pneumatic DIW 3D printer (Bioscaffolder 3.1, GeSiM, Germany) was used to fabricate the fractal-like scaffolds by calling the customized fabrication G-codes (*.nc file). Different inks were extruded by the 3D printer along with the designed pattern. For the DIW 3D printed PLGA scaffolds, the inner diameter of the nozzle was 260 μm; the moving speed of the nozzle was 540 mm min^-1^; the air pressure of the nozzle was ~400 to 500 kPa; and the temperature of the additional low-temperature platform (FP50-ME, Julabo, Germany) was set to -35°C. The PLGA ink was deposited on the low-temperature platform to build predesigned samples. The obtained low-temperature PLGA samples were quickly transported to the low-temperature vacuum drying oven and processed at -80°C for 72 hours. For the DIW 3D printed β-TCP/PCL scaffolds, the inner diameter of the nozzle was 350 μm; the moving speed of the nozzle was 480 mm/min; the air pressure of the nozzle was ~340 kPa; the printing temperature of the nozzle was ~70℃. For the DIW 3D printing of the GelMA/Alg/hMSCs bioink, the inner diameter of the nozzle was 410 μm; the moving speed of the nozzle was 300 mm min^-1^; the air pressure of the nozzle was ~40 kPa. The 3D printed GelMA/Alg/hMSCs scaffolds were post-processed with UV light irradiation for 5-10 seconds. For the DIW 3D printing of the pure dye/Alg ink, the inner diameter of the nozzle was 350 μm; the moving speed of the nozzle was 300 mm min^-1^; the air pressure of the nozzle was ~360 kPa.

**Cell viability**

The live/dead of hMSCs on the bioprinted fractal-like scaffolds was stained with fluorescein diacetate/propidium iodide (FDA/PI) for 10 min and then observed by a confocal laser scanning microscope (CLSM, Leica TCS SP8, Wetzlar, Germany).

**Micro-CT analysis and porosity characterization**

Four samples (n = 4) of each type of the 3D printed PLGA scaffolds were scanned by a high-resolution scanning system (SkyScan 1176, Bruker, USA). The setting parameters for this work were 40 kV for x-ray tube voltage, 596 μA for x-ray tube current, no filter for the optical filter, 9 μm for a pixel size of spatial resolution, 0.6° for rotation step, and 360° for scanning angular scope. We carried out an evaluation and three-dimensional reconstruction of the PLGA scaffold via post-processing software of the company Bruker according to the following steps: (i) converting the micro-CT images obtained by SkyScan 1176 into a grayscale image of 0-255 via software NRecon, (ii) making the grayscale image horizontal and vertical via software DataViewer, (iii) calculating the cylindrical porosity and the SS/SV of the global and local scaffolds based on the horizontal and vertical images via software CTAn, and (iv) obtaining the reconstructed models via software CTvox (Figure 5(IV)-4(VI)). Limited by the underlying algorithm of CTAn software, the hollow center area (mimic marrow cavity) of the hollow cylindrical models should be removed when calculating the porosity, otherwise, it will be larger than the actual value. The global (Equation 1) and local (Equation 2) porosity (*ϕ*_Sample_ and *ϕ_i_*) of the hollow cylindrical models are shown in the following equations.

where *ϕ*_Sample_ is the porosity of the global hollow cylindrical samples excluding the hollow center area, *ϕ*_Cylindrical_ is the porosity of the cylindrical samples including the hollow center area and can be obtained from software CTAn, *r*_Outer_ and *r*_Inner_ are the outer and inner radius of the hollow cylinder constructed from the ROI of the femur respectively, and *π* is for Pi.

where *ϕ*_i_ is the porosity of the *i*-th zone of the local hollow cylindrical samples equally divided from the inner to the outer zone, *i* represents the number of the equally spaced zone (*i* = iterations×2), and its values is an integer starting from 1, ϕ_Zone_ is the porosity of the cylindrical samples with an inner diameter of (*r*_Inner_+(*i*-1)×*r*_d_) and an outer diameter of (*r*_Inner_+*i*×*r*_d_) based on Boolean operations and can be obtained from software CTAn, *r*_d_ is the step size for the equally spaced zone (*r*_d_ = (*r*_Outer_ - *r*_Inner_)/i), and *r*_Outer_, *r*_Inner_, and *π* share the definition of the Equation 1. The height H of the scaffolds is removed from the top and bottom of the formula, so it is not displayed (Equations 1 and 2).

**Uniaxial compression testing**

Four samples (n = 4) of each type of the 3D printed PLGA scaffolds were tested. Uniaxial compression tests were performed on a ZwickRoell testing machine using a mechanical sensor of 5 kN and a compression speed of 1 mm min^-1^ up to a strain of 50%. The compressive modulus was calculated as the slope of the initial linear segment of the stress-strain curve.

**Finite element modeling of the uniaxial compression**

To facilitate the uniaxial compression simulation, each type of the designed porous scaffold models of scaffolds was cut with one-tenth of the filament diameter in the upper and lower regions of the cylindrical section and then merged into a continuous part through Boolean intersection operation. The obtained part was exported into Abaqus 6.14 (Dassault System Simulia Corp, Providence, RI, USA) as Parasolid (*.x_t) files. A 10-node modified quadratic tetrahedron (C3D10M) was applied to mesh the part. To reduce the fluctuation of the numerical results, we refined the sufficient number of elements on the finite element models of porous scaffolds. The conventional lay-down pattern of 0°/90°, 0-iterations fractal-like structures, and 3-iterations fractal-like structures have 1,891,040, 2,255,850, and 2,504,140 elements, respectively. The implicit static module of the software was implemented to simulate uniaxial compression. The bottom surface of the scaffold model was fixed, and its top surface was given a 10% strain for linear FEA simulation (Figure 6(A)). In the simulation, PLGA is considered to be homogeneous and isotropic linear elastic. The Poisson’s ratio and Young’s modulus are 0.3 and 2.0 GPa [[2](#_ENREF_2)], respectively. The equivalent modulus of the porous scaffold is calculated in the following equation

$$E_{\text{S}}\text{ = }\frac{\sigma}{\varepsilon}\text{ }=\text{ }\frac{F/S}{\Delta h/H}$$

where *E*_S_ (MPa) is the equivalent modulus, *σ* (N m^-2^) is the stress, *ε* (m m^-1^) is the strain, *F* (N) is the force, *S* (m^2^) is the axial cross-sectional area of the hollow cylinder, Δ*h* (m) is the scaffold height difference between before and after uniaxial compression, and *H* (m) is the height of scaffold before compression.

**Permeability testing**

In this work, we used the falling head method and the variable head method to measure the axial and radial permeability of hollow cylindrical samples, respectively. The axial and radial permeability setups (see Figure S15 and schematic diagrams shown in Figure 7(g) and 7(h)) were designed and fabricated. Dulbecco’s modified Eagle’s medium (DMEM) was used as a medium for fluid experiments. The effective axial (*K*_Axial_, Equation 4) and radial (*K*_Radial_, Equation 5) permeability of the samples could be calculated by Darcy's law as shown in the following equations [[3](#_ENREF_3), [4](#_ENREF_4)].

$$K_{\text{Axial}}=\frac{a}{A}\frac{h}{t}\ln\frac{H_{1}}{H_{2}}\cdot\frac{\mu}{\rho g}$$

where *K*_Axial_ (m^2^) is the effective axial permeability, *a* (m^2^) is the cross-sectional area of the standpipe, *A* (m^2^) is the cross-sectional area of the hollow cylindrical scaffolds excluding the hollow center area, *h* (m) is the height of the scaffolds, *t* (s) is the experiment time, *H*_1_ (m) and *H*_2_ (m) are the DMEM level at the beginning and ending of the experiment, *μ* (Pa s) and *ρ* (kg m^-3^) are the dynamic viscosity and the density of DMEM respectively, *g* (m s^-2^) represents the gravitational acceleration, and ln represents the natural logarithm with e as the base.

In the axial permeability tests, a solid pin (Figure S15 A(6)) was placed in the hollow center area of the hollow cylindrical samples (Figure S15 A(7)) to prevent DMEM from passing through this area (Figure 7(h)). We first turned off the switch of the water outlet (Figure S15 A(10)), cleared the stopwatch (Figure S15 A(12)), and then poured DMEM into the standpipe (Figure S15 A(1)) and recorded the height of the current liquid level in the stopwatch from the liquid level of the overflow trough (Figure S15 A(11)) as *H*_1_. Then, we started the experiment, turned on the switch of the water outlet (Figure S15 A(10)) and the stopwatch (Figure S15 A(12)) at the same time; after *t*-time experiment, quickly turned off water outlet (Figure S15 A(10)) and the stopwatch (Figure S15 A(12)), recorded the current height of the liquid level in the standpipe (Figure S15 A(1)) from the liquid level of the overflow trough (Figure S15 A(11)) as *H*_2_, and recorded the stopwatch (Figure S15 A(12)) time as *t*. After the experiment was completed, we also record the inner diameter (*D*_Inner_), outer diameter (*D*_Outer_), and height dimensions (*h*) of the sample (Figure S15 A(7)) for calculating the axial permeability via Darcy's law (*K*_Axial_, Equation 4).

$$K_{\text{Radial}}\text{=}\frac{V}{t}\frac{\mu\ln\left(r_{\text{Inner}}/r_{\text{Outer}}\right)}{2\pi h\left(P_{\text{Inner}}-P_{\text{Outer}}\right)}$$

where *K*_Radial_ (m^2^) is the effective radial permeability, *V* (m^3^) is the volume of DMEM passing through the scaffolds during the experiment, *t* (s) is the experiment time of collecting DMEM in the beaker, *μ* (Pa s) is the dynamic viscosity, *r*_Inner_ (m) and *r*_Outer_ (m) are the inner and outer radius of the scaffolds, *h* (m) is the height of the scaffolds, *P*_Inner_ (m) and *P*_Outer_ (m) are the inlet and outlet pressure of the scaffolds at the radial permeability setup, and *π* is for Pi.

In the radial permeability tests, we first turned off the switch of the water outlet (Figure S15 B(7)), cleared the stopwatch (Figure S15 B(9)), turned on the switch of the water inlet (Figure S15 B(10)), and poured DMEM into the standpipe (Figure S15 B(1)). Then we turned on the switch of the water outlet (Figure S15 B(7)). After the experiment entered a steady-state (Figure S15 B(2) and B(6)), we recorded the height of the liquid level respectively. In addition, the volume (*V*) of DMEM (Figure S15 B(8)) was collected in the beaker (*t*) within the time recorded by the stopwatch (Figure S15 B(9)). After the experiment was completed, record the height of the sample (Figure S15 B(3)) for calculating the axial permeability.

**Computation fluid dynamic of the permeability**

CFD simulation was calculated in software COMSOL using a laminar and steady-state Navier-Stokes model. The permeability simulations were performed along the radial and axial directions of the scaffolds. The Navier-Stokes model includes the conservation of momentum equation (Equation 6) and the conservation of mass equation (Equation 7).

$$\rho\left(\mathbf{u}\cdot\nabla\right)\mathbf{u}=\nabla\cdot\left\{-p\mathbf{I}+\mu\left[\nabla\mathbf{u}+\left(\nabla\mathbf{u}\right)^{\text{T}}\right]\right\}+\mathbf{F}$$

$$\rho\nabla\cdot\left(\mathbf{u}\right)=0$$

where *ρ* (kg m^-3^) is the fluid density, **u** (m s^-1^) is the fluid velocity, ∇ is a mathematical operator that represents the gradient, *p* (Pa) is the pressure, **I** is the identity matrix, μ (Pa s) is the fluid dynamic viscosity, T means transpose, **F** is the external force exerted on the fluid.

The scaffold model obtained by Grasshopper was imported into COMSOL Multiphysics 5.4 as IGES format. In software COMSOL, the hollow cylinder (inner diameter = 4.5 mm, outer diameter = 10.5 mm, height = 4 mm) and the scaffold model (inner diameter = 5 mm, outer diameter = 10 mm, height = 4 mm) were subjected to Boolean subtraction to obtain the fluid flow computation domain (Figure S12, Supporting Information). In the computation fluid dynamics, the laminar flow and the steady-state Navier-Stokes model were implemented. The inner and outer walls of the computation domain were defined as the inlet (flow rate, *v*_i_ = 1 mm s^-1^) and outlet (pressure, *P* = 0 Pa), respectively. The remaining walls of the computation domain were set to be non-slip. The reference temperature was considered as the human body temperature of 37°C. Effective permeability is calculated by Darcy's law, as shown in the following equation

$$K\text{ = }v\times\frac{\mu L}{\Delta P}$$

where *K* (m^2^) is the effective permeability, *v* (mm s^-1^) is Darcy’s velocity, *μ* (Pa s) is the dynamic viscosity, *L* (m) is the domain length, and Δ*P* is the pressure difference between the inlet and the outlet of the computation domain. Among them, the length of the computation domain was L = 7 mm. *v* and Δ*P* could be obtained by COMSOL simulation. Dulbecco’s modified Eagle’s medium (DMEM) as a liquid medium was used to simulate the effective permeability of the scaffold. This medium was considered as incompressible, continuous, and isotropic Newtonian fluid. The selected properties of the DMEM were dynamic viscosity, *μ* = 1.45×10^-3^ Pa s; density, *ρ* = 1000 kg m^-3^ [[5](#_ENREF_5)].

**Code availability**

The customized battery for designing the novel fractal-like scaffolds, the GhPython codes for converting the fractal-like model to fabrication codes, and additional details used in the work can be obtained from the corresponding authors.

**Statistical analysis**

All data were reported as mean ± SD (standard deviation). One-way ANOVA and Tukey’s posthoc test were implemented using Origin 2021 (Learning Edition) software (OriginLab Corporation, USA). Differences in statistical analyses were considered significant when **P* < 0.05, ***P* < 0.01, ****P* < 0.001. n.s. means no significant difference.

**References**

1. D. Loessner, C. Meinert, E. Kaemmerer, L. C. Martine, K. Yue, P. A. Levett, T. J. Klein, F. P. W. Melchels, A. Khademhosseini, D. W. Hutmacher, "Functionalization, preparation and use of cell-laden gelatin methacryloyl–based hydrogels as modular tissue culture platforms," *Nature Protocols,* vol. 11, no. 4, pp. 727-746, 2016.

2. P. Gentile, V. Chiono, I. Carmagnola, V. P. Hatton, "An overview of poly(lactic-co-glycolic) acid (PLGA)-based biomaterials for bone tissue engineering," *International Journal of Molecular Sciences,* vol. 15, no. 3, pp. 3640-3659, 2014.

3. J. Amyx, D. Bass, R. L. Whiting, *Petroleum reservoir engineering physical properties* (McGraw-Hill Book Company, Inc., New York, 1960).

4. X. Y. Zhang, G. Fang, S. Leeflang, A. A. Zadpoor, J. Zhou, "Topological design, permeability and mechanical behavior of additively manufactured functionally graded porous metallic biomaterials," *Acta Biomaterialia,* vol. 84, pp. 437-452, 2019.

5. S. Gomez, M. D. Vlad, J. Lopez, E. Fernandez, "Design and properties of 3D scaffolds for bone tissue engineering," *Acta Biomaterialia,* vol. 42, no. 2016, pp. 341-350, 2016.

6. H. Montazerian, M. G. A. Mohamed, M. M. Montazeri, S. Kheiri, A. S. Milani, K. Kim, M. Hoorfar, "Permeability and mechanical properties of gradient porous PDMS scaffolds fabricated by 3D-printed sacrificial templates designed with minimal surfaces," *Acta Biomaterialia,* vol. 96, pp. 149-160, 2019.

7. N. Soro, N. Saintier, J. Merzeau, M. Veidt, M. S. Dargusch, "Quasi-static and fatigue properties of graded Ti–6Al–4V lattices produced by Laser Powder Bed Fusion (LPBF)," *Additive Manufacturing,* vol. 37, pp. 101653, 2021.

8. E. D. Sanders, A. Pereira, G. H. Paulino, "Optimal and continuous multilattice embedding," *Science Advances,* vol. 7, no. 16, pp. eabf4838, 2021.

9. S. J. P. Callens, C. H. Arns, A. Kuliesh, A. A. Zadpoor, "Decoupling Minimal Surface Metamaterial Properties Through Multi-Material Hyperbolic Tilings," *Advanced Functional Materials,* vol. 31, no. 30, pp. 2101373, 2021.

10. X. J. Wang, S. Q. Xu, S. W. Zhou, W. Xu, M. Leary, P. Choong, M. Qian, M. Brandt, Y. M. Xie, "Topological design and additive manufacturing of porous metals for bone scaffolds and orthopaedic implants: A review," *Biomaterials,* vol. 83, no. 2016, pp. 127-141, 2016.

11. C. J. Han, C. Z. Yan, S. F. Wen, T. Xu, S. Li, J. Liu, Q. S. Wei, Y. S. Shi, "Effects of the unit cell topology on the compression properties of porous Co-Cr scaffolds fabricated via selective laser melting," *Rapid Prototyping Journal,* vol. 23, no. 1, pp. 16-27, 2017.

12. A. Clausen, F. Wang, J. S. Jensen, O. Sigmund, J. A. Lewis, "Topology Optimized Architectures with Programmable Poisson's Ratio over Large Deformations," *Advanced Materials,* vol. 27, no. 37, pp. 5523-5527, 2015.

13. S. Nachtrab, S. C. Kapfer, C. H. Arns, M. Madadi, K. Mecke, G. E. Schroder-Turk, "Morphology and linear-elastic moduli of random network solids," *Advanced Materials,* vol. 23, no. 22-23, pp. 2633-+, 2011.

14. M. Z. Liang, G. D. Zhang, F. Y. Lu, X. Y. Li, "Blast resistance and design of sandwich cylinder with graded foam cores based on the Voronoi algorithm," *Thin-Walled Structures,* vol. 112, pp. 98-106, 2017.

15. M. Fantini, M. Curto, F. De Crescenzio, "A method to design biomimetic scaffolds for bone tissue engineering based on Voronoi lattices," *Virtual And Physical Prototyping,* vol. 11, no. 2, pp. 77-90, 2016.

16. A. Entezari, I. Roohani, G. L. Li, C. R. Dunstan, P. Rognon, Q. Li, X. Q. Jiang, H. Zreiqat, "Architectural design of 3D printed scaffolds controls the volume and functionality of newly formed bone," *Advanced Healthcare Materials,* vol. 8, no. 1, pp. 12, 2019.

17. S. M. Giannitelli, D. Accoto, M. Trombetta, A. Rainer, "Current trends in the design of scaffolds for computer-aided tissue engineering," *Acta Biomaterialia,* vol. 10, no. 2, pp. 580-594, 2014.

18. J. L. Davila, M. S. Freitas, P. I. Neto, Z. C. Silveira, J. V. L. Silva, M. A. d'Avila, "Fabrication of PCL/beta-TCP scaffolds by 3D mini-screw extrusion printing," *Journal of Applied Polymer Science,* vol. 133, no. 15, pp. 9, 2016.

19. R. Comminal, M. P. Serdeczny, D. B. Pedersen, J. Spangenberg, "Motion planning and numerical simulation of material deposition at corners in extrusion additive manufacturing," *Additive Manufacturing,* vol. 29, pp. 17, 2019.

20. M. P. Serdeczny, R. Comminal, D. B. Pedersen, J. Spangenberg, "Numerical simulations of the mesostructure formation in material extrusion additive manufacturing," *Additive Manufacturing,* vol. 28, pp. 419-429, 2019.

21. M. P. Serdeczny, R. Comminal, D. B. Pedersen, J. Spangenberg, "Experimental validation of a numerical model for the strand shape in material extrusion additive manufacturing," *Additive Manufacturing,* vol. 24, pp. 145-153, 2018.

22. H. M. He, D. Li, Z. F. Lin, L. Q. Peng, J. Yang, M. M. Wu, D. L. Cheng, H. B. Pan, C. S. Ruan, "Temperature-programmable and enzymatically solidifiable gelatin-based bioinks enable facile extrusion bioprinting," *Biofabrication*, 2020.

23. E. Davoodi, E. Sarikhani, H. Montazerian, S. Ahadian, M. Costantini, W. Swieszkowski, S. M. Willerth, K. Walus, M. Mofidfar, E. Toyserkani, A. Khademhosseini, N. Ashammakhi, "Extrusion and Microfluidic-Based Bioprinting to Fabricate Biomimetic Tissues and Organs," *Advanced Materials Technologies*, pp. 30.

24. N. E. Putra, M. A. Leeflang, M. Minneboo, P. Taheri, L. E. Fratila-Apachitei, J. M. C. Mol, J. Zhou, A. A. Zadpoor, "Extrusion-based 3D printed biodegradable porous iron," *Acta Biomaterialia,* vol. 121, pp. 741-756, 2021.

25. Z. Jiang, B. Diggle, M. L. Tan, J. Viktorova, C. W. Bennett, L. A. Connal, "Extrusion 3D Printing of Polymeric Materials with Advanced Properties," *Advanced Science,* vol. 7, no. 17, pp. 2001379, 2020.

26. S. D. Lacey, D. J. Kirsch, Y. Li, J. T. Morgenstern, B. C. Zarket, Y. Yao, J. Dai, L. Q. Garcia, B. Liu, T. Gao, S. Xu, S. R. Raghavan, J. W. Connell, Y. Lin, L. Hu, "Extrusion-Based 3D Printing of Hierarchically Porous Advanced Battery Electrodes," *Advanced Materials,* vol. 30, no. 12, pp. 1705651, 2018.


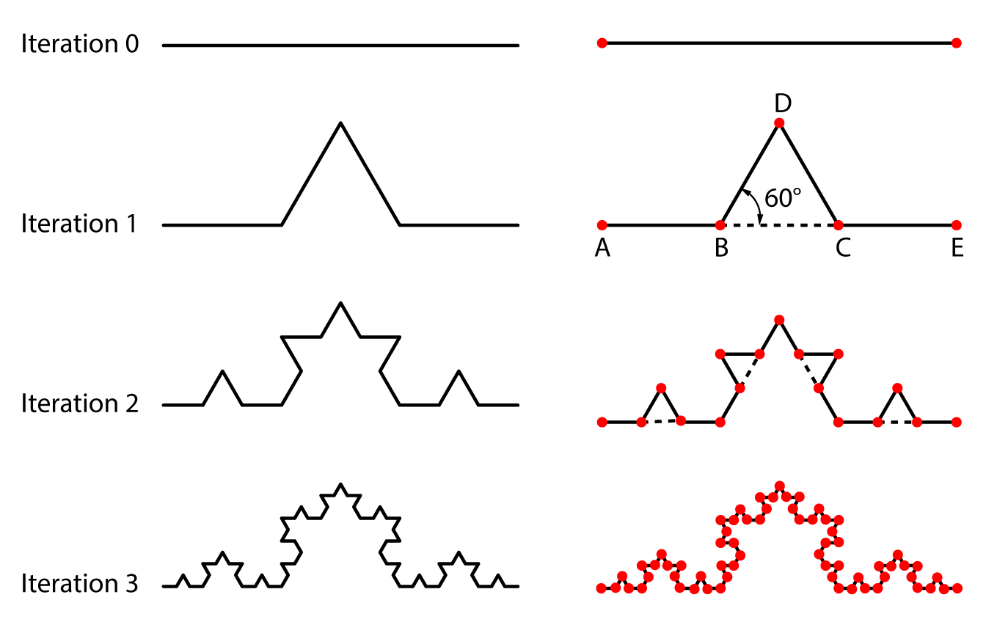


**Figure S1.** Four iteration stages of the Koch snowflake with 0, 1, 2, and 3 iterations. A line segment is divided into three equal parts, and four points (Points A, B, C, and E) of each line segment are obtained. The third point (Point C) is rotated 60° counterclockwise around the second point (Point B), and these points (Point A, B, D, C, and E) are connected in sequence to obtain a Koch snowflake with 1 iteration.


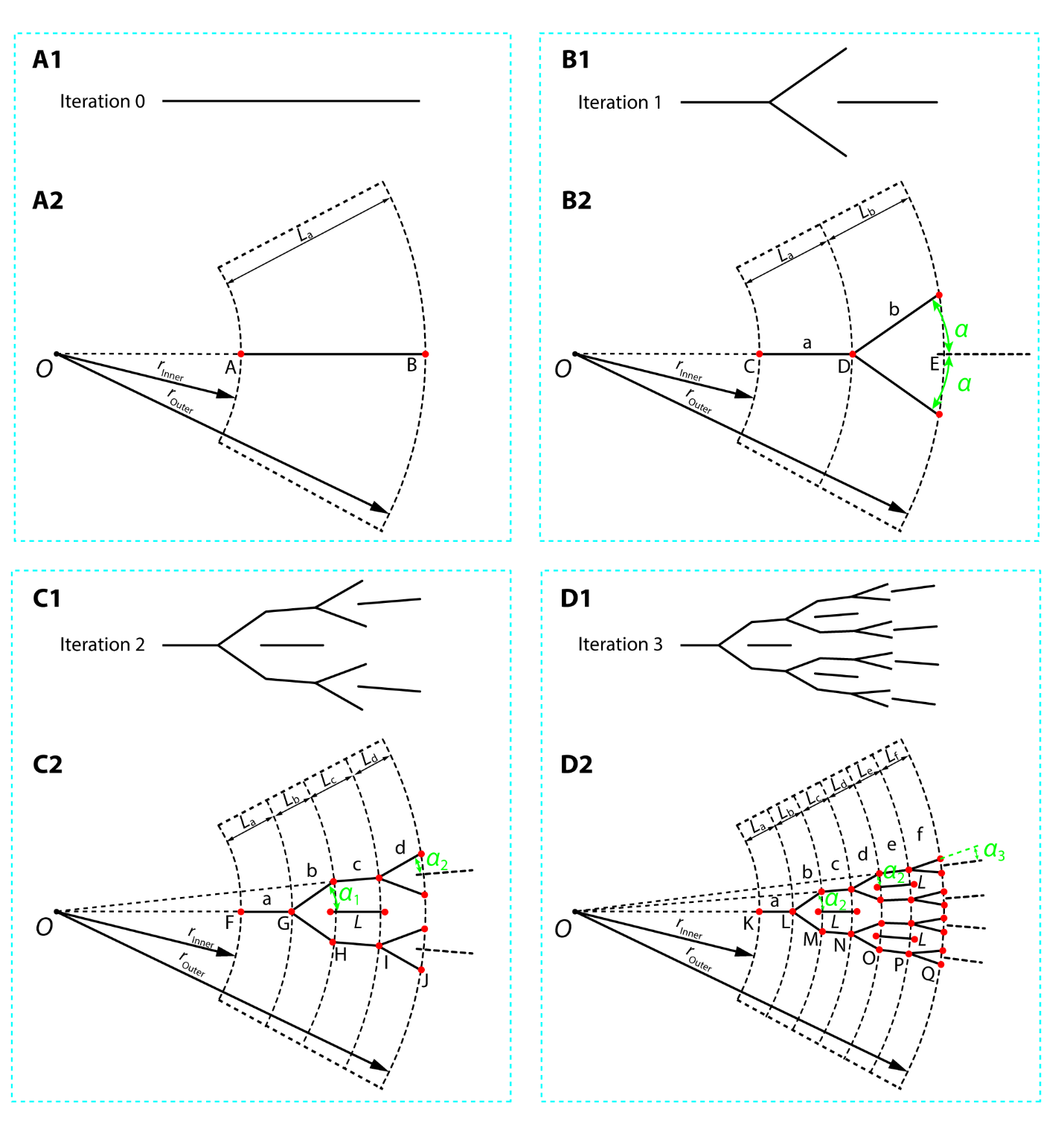


**Figure S2.** Four iteration stages of 2D fractal-like curves with 0, 1, 2, and 3 iterations. (A1), (B1), (C1), and (D1) show the results of the 2D fractal-like tree curves with 0, 1, 2, and 3 iterations, respectively. These curves are symmetrical. (A2), (B2), (C2), and (D2) respectively show the rules of trimming the above-mentioned four 2D fractal curves and designing 2D concentric ring layer curves based on branch points (red dot in the picture) of the trimmed curve. In this rule, the outermost tail segments of the 2D fractal curves were removed. The start points and the end points of the trimmed fractal curve are placed on the inner wall and the outer wall of the target tissue substitute, respectively. The odd-numbered line segments of the trimmed fractal curve are all along the radial direction. The fractal-like scaffolds with 0, 1, 2, and 3 iterations have 0, 1, 2, and 3 level branching angles (*α*), respectively.


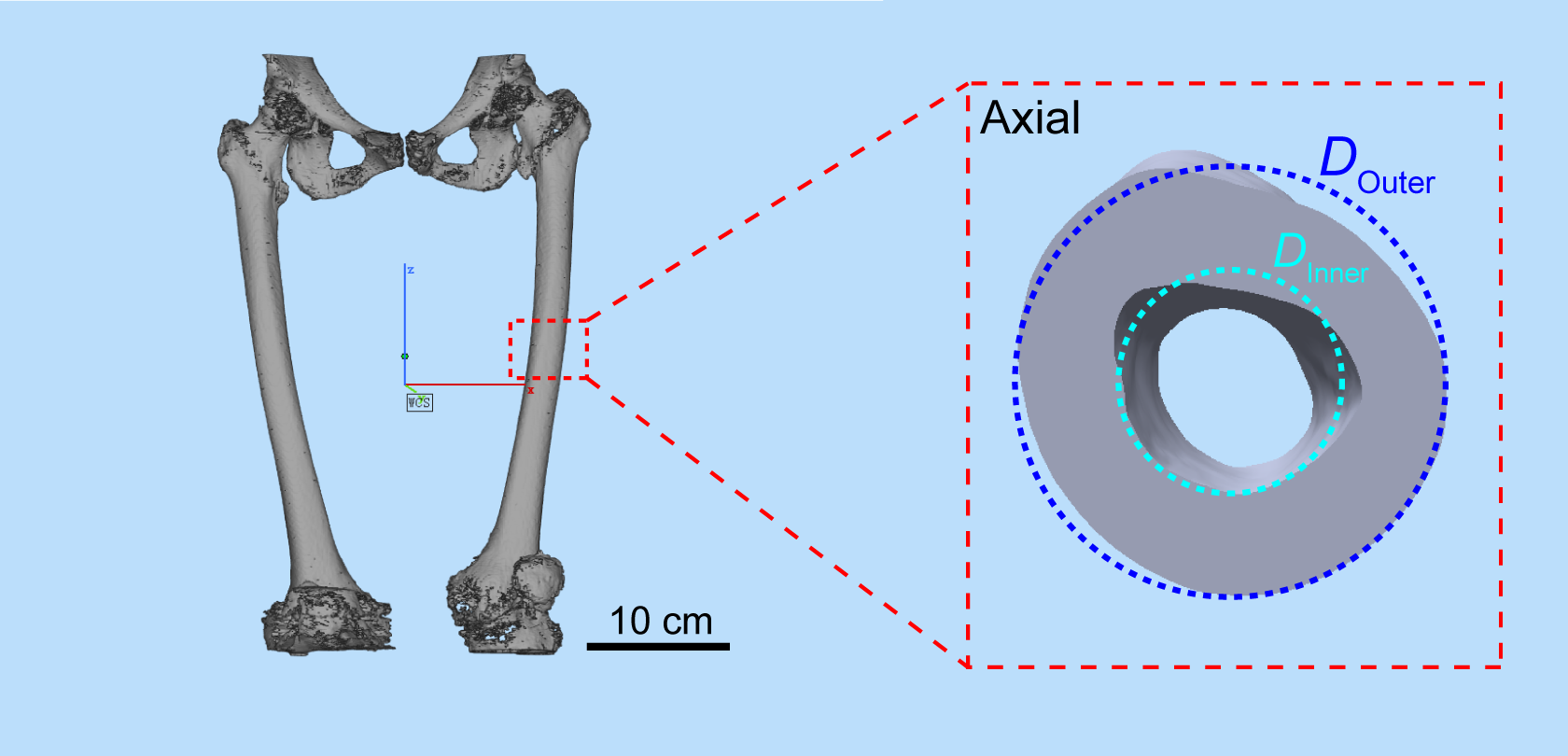


**Figure S3.** CT data a 58-year-old Chinese woman femur. The use of CT data was licensed.


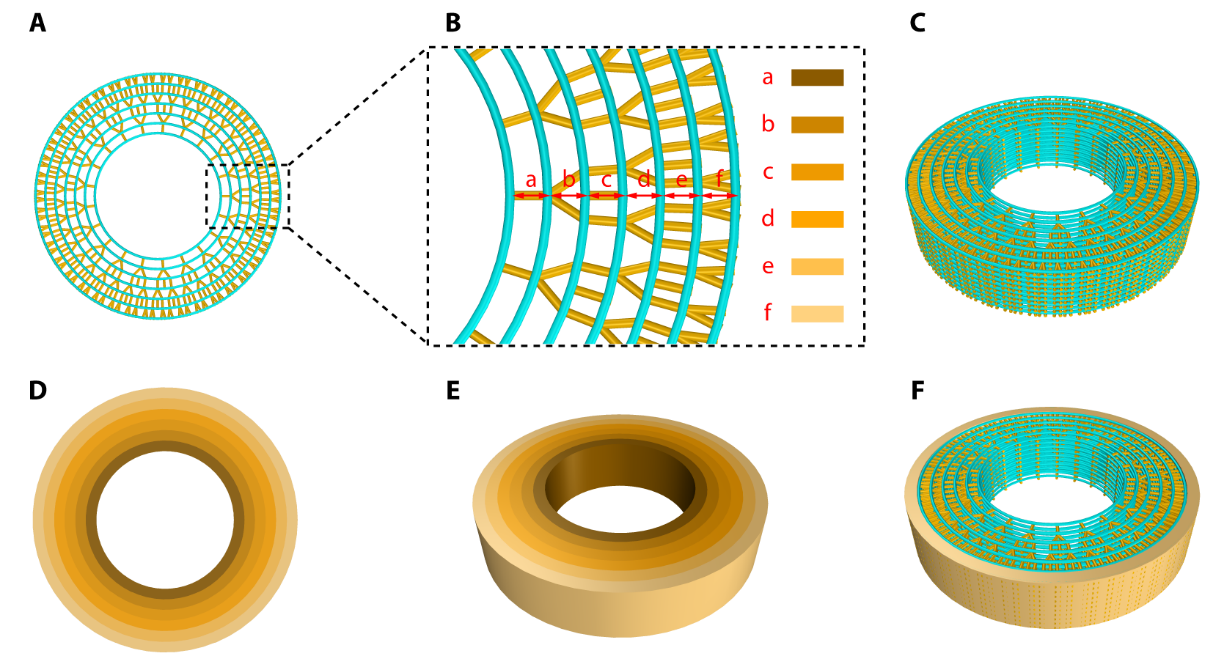


**Figure S4.** Definition of the local zones of the porous scaffolds in the radial direction for evaluating the local porosity and the local SS/SV. After removing the innermost circle of the ring layer, the scaffolds were divided into six regions along the radial direction, respectively named as zones a, b, c, d, e, and f. (A) Top view of the fractal-like scaffold with 3 iterations. (B) Magnified view of the scaffold divided into six radial sections. (C) Axonometric drawing of the scaffold. (D) Top view of the six radial zones. (E) Axial diagram of the six radial zones. (F) Axonometric drawing of the zone f by the Boolean operation.


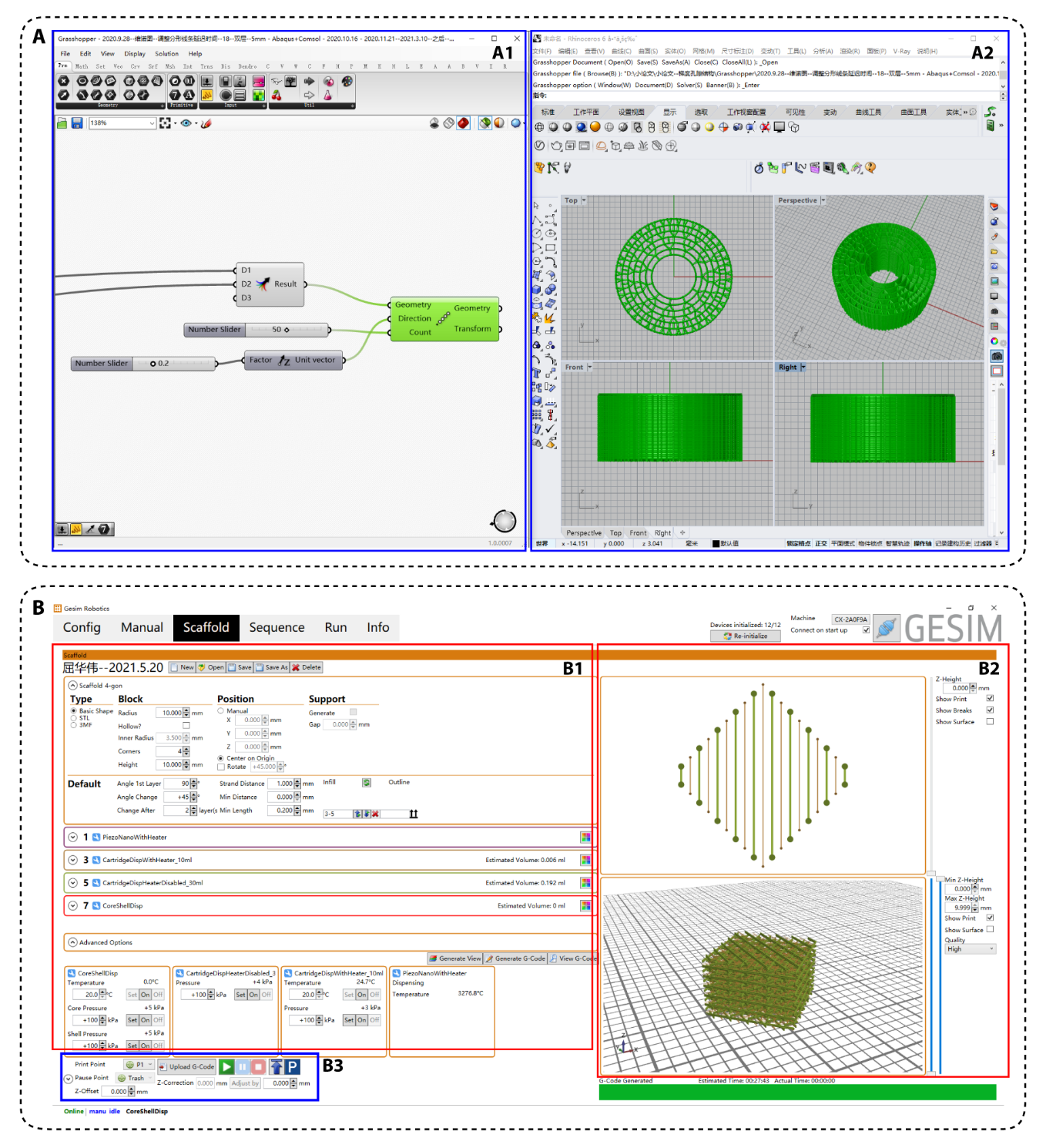


**Figure S5.** Establishing the software implementations of the design-to-fabrication workflow from the designed CAD models to the EB-3D printed samples. (A) Design of the fractal-like scaffolds and programming of the fabrication codes (G-codes). Realization of the digital workflow of the fractal-like scaffolds was carried out in the visual programming software Grasshopper, as a plug-in of Rhinoceros software. Grasshopper software interface (A1) and Rhinoceros software interface (A2) were used to design models and display the designed models in real-time, respectively. (B) DIW 3D printing of the scaffolds implement in the software GeSiM Bioscaffolder 3.1 by updating and calling the obtained G-codes. The print parameter setting interface (B1) and model display interface (B2) in software GeSiM Bioscaffolder 3.1 were not adopted, and they were replaced by Grasshopper software interface (A1) and Rhinoceros software interface (A2) respectively. The fabrication codes (G-codes) obtained in the parametrically designed model can be updated and read by the GeSiM printer software to control the movement of the printhead to deposit ink along the required path (B3). In this figure, the part in the red rectangle was discarded, but the part in the green rectangle was needed.


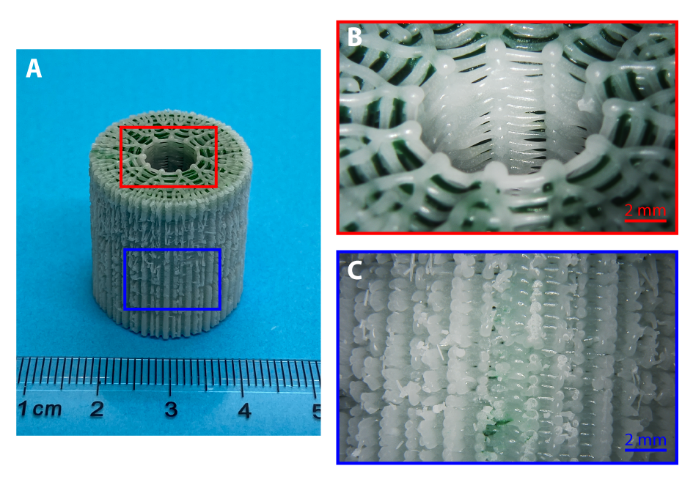


**Figure S6.** Axonometric (A) and enlarged (B and C) views of the 3D bio-printed fractal-like scaffolds with 3 iterations.


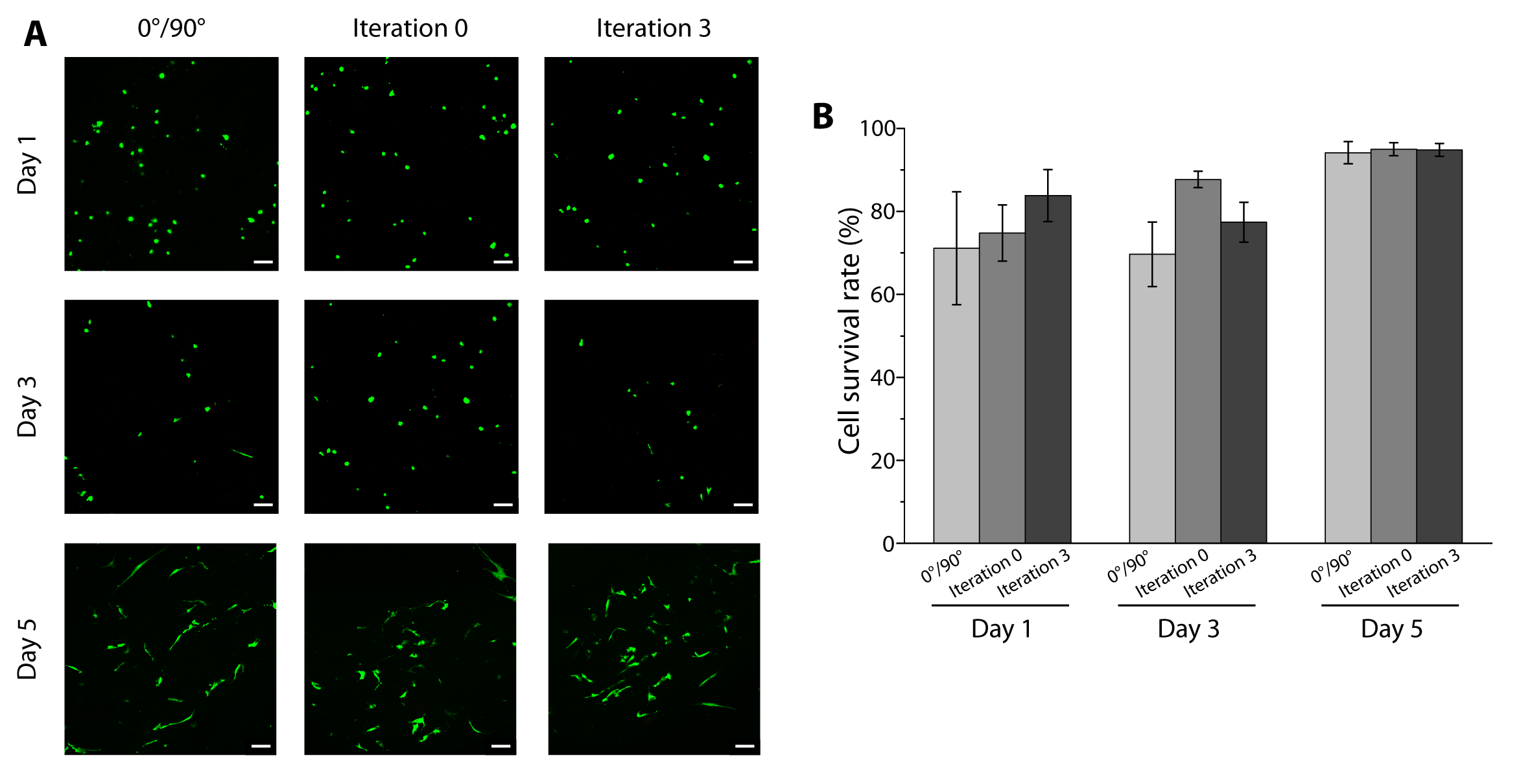


**Figure S7.** Qualitative (A) and quantitative (B) analysis of live/dead staining for the bio-printed porous scaffolds on days 1, 3, and 5. Scale bars, 100 µm.


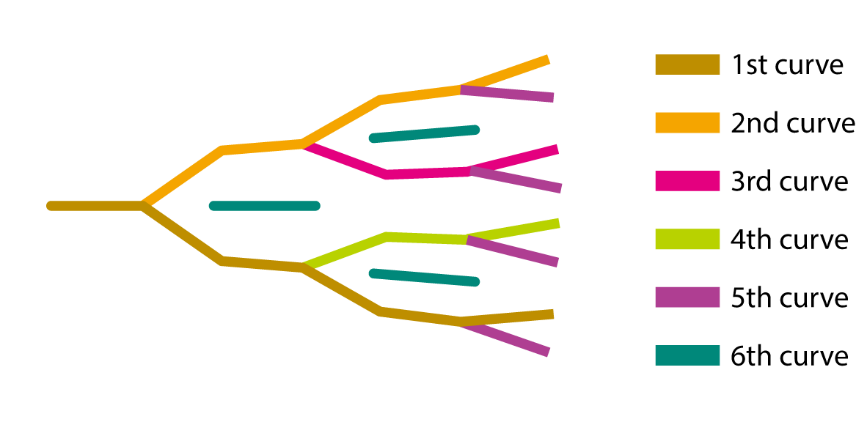


**Figure S8.** Deposition sequence of the 2D fractal curves of the fractal-like scaffolds. The dynamic display of the deposition can be found in movies S4 and S5.


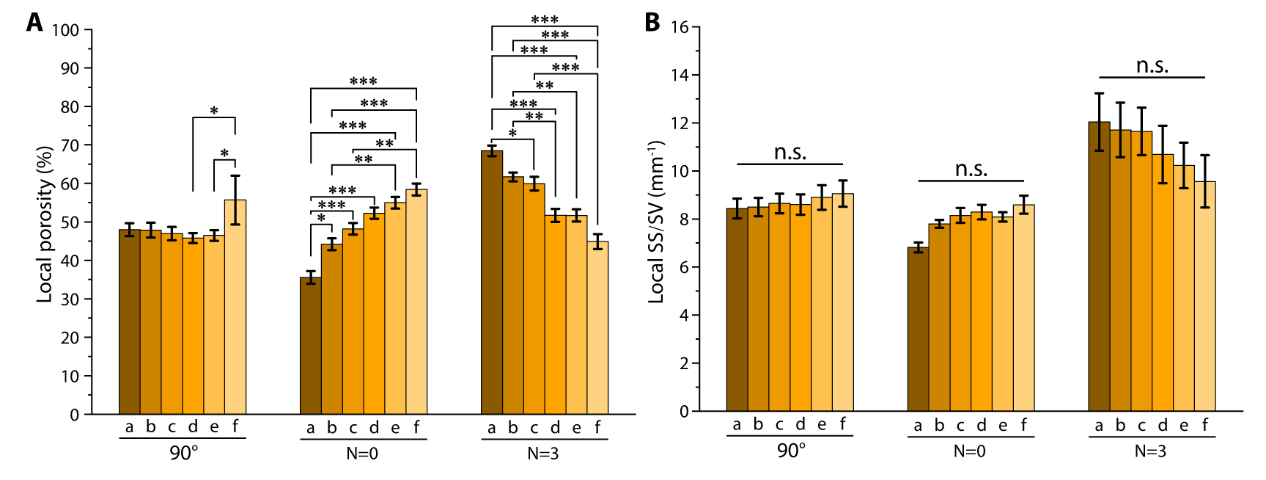


**Figure S9.** Quantification of local porosity and local SS/SV of porous scaffolds in the radial direction. Differences in statistical analyses were considered significant when **P* < 0.05, ***P* < 0.01, ****P* < 0.001. n.s. means no significant difference.


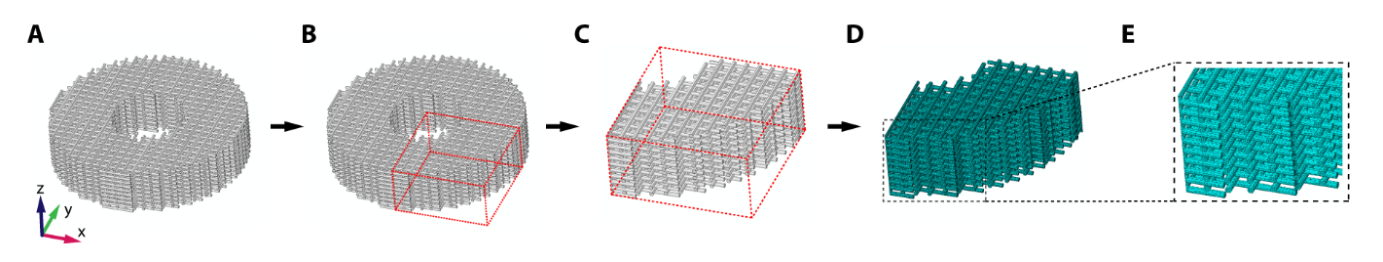


**Figure S10.** Main steps for converting CAD models into finite element meshes of the uniaxial compression. (A) A CAD model with a lay-down pattern of 0°/90° (Inner diameter = 8 mm, outer diameter = 22 mm, and height = 3 mm). (B) Selecting a quarter of the model as the actual computational domain due to the symmetry of the model. (C) Obtaining the actual computational domain. (D) Meshing the domain. (E) Partial enlarged view of the domain. All steps are operated in Abaqus 6.14.


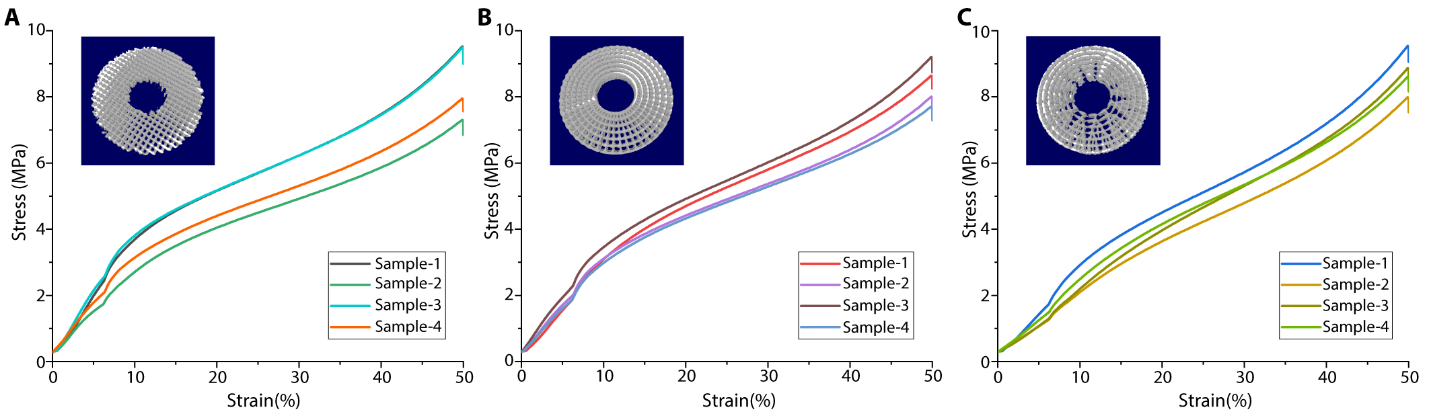


**Figure S11.** Uniaxial compression tests of the porous scaffolds. (A), (B), and (C) represent the scaffolds with a lay-down pattern of 0°/90°, 0 iterations, and 3 iterations respectively. Four samples (n = 4) of each type of scaffold were tested.


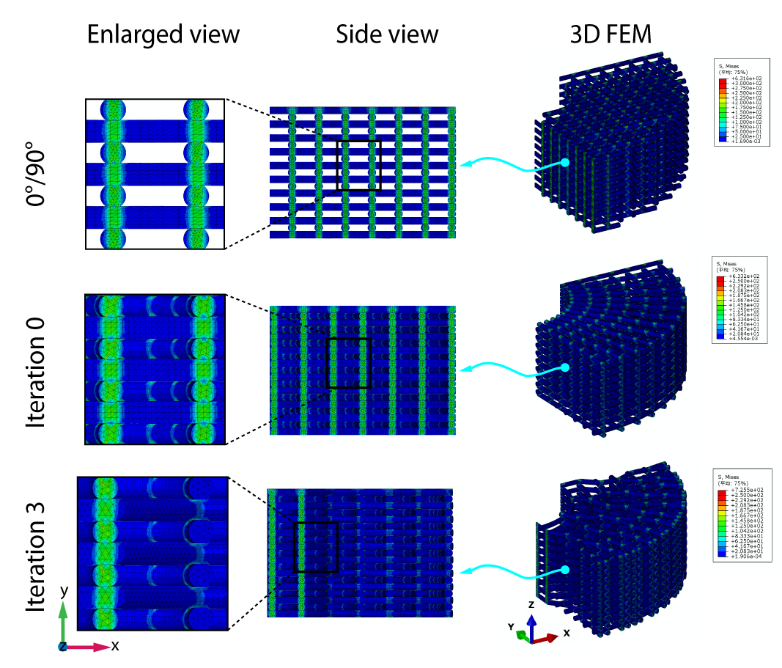


**Figure S12.** FEA simulation of uniaxial compression. Side view and enlarged view of the FEA simulation of the three scaffolds with a lay-down pattern of 0°/90°, 0 iterations, and 3 iterations.


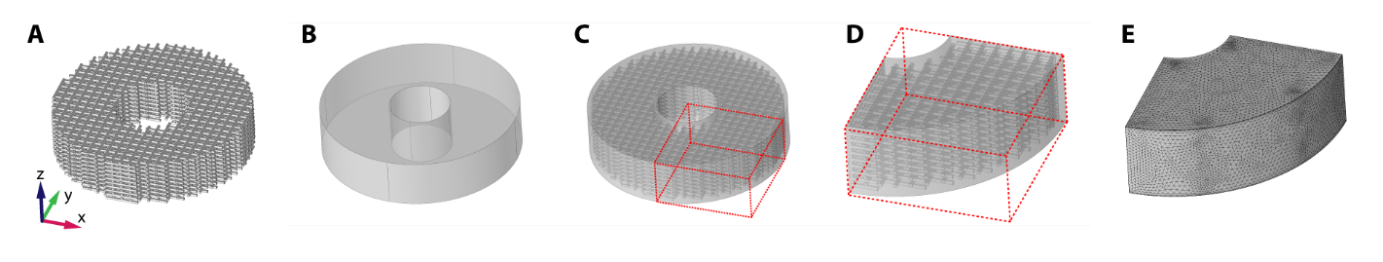


**Figure S13.** Main steps for converting CAD models into fluid computation domains for the radial and axial permeability measurements. (A) A CAD model with a lay-down pattern of 0°/90° (Outer diameter is 22 mm, inner diameter is 8 mm, and height is 5 mm). (B) A hollow cylinder domain containing the CAD model (Outer diameter is 23 mm, inner diameter is 7 mm, and height is 6.5 mm). (C) The fluid computation domain obtained by Boolean subtraction of the domain (B) and the CAD model (A). (D) Obtaining the actual computational domain from a quarter of the overall due to the symmetry of the model. (E) Meshing the computational domain. All steps are operated in COMSOL Multiphysics 5.4.


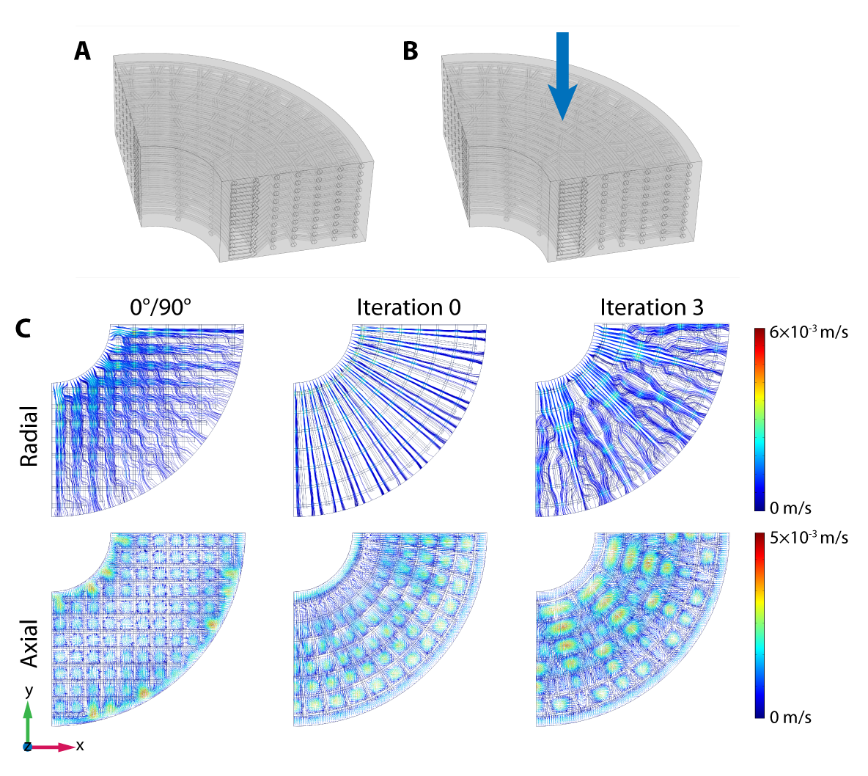


**Figure S14.** Top view of CFD simulation. (A) Selected fluid simulation domain. (B) Schematic diagram showing the direction of the top view. (C) Top view of the radial and axial fluid flow simulation results of the three scaffolds with a lay-down pattern of 0°/90°, 0 iterations, and 3 iterations.


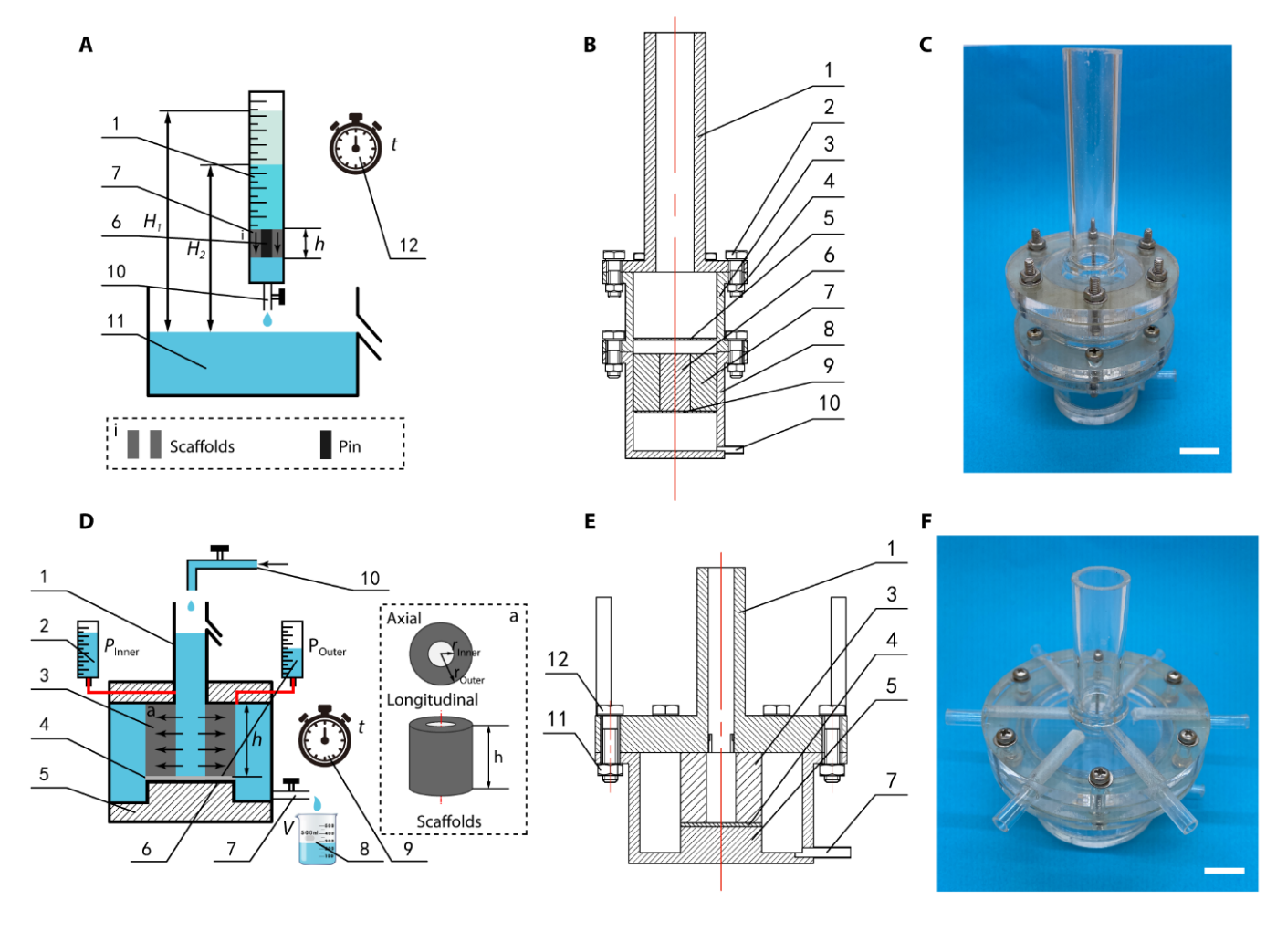


**Figure S15.** Schematic diagrams (A and D), assembly drawings (B and E), and main pictures (C and F) of the axial and radial permeability setups. The axial (A to C) and radial (D to F) permeability setups were implemented using the falling head method and the constant head method, respectively. The setups were manufactured by using acrylic material as the raw material. For the axial permeability setup (A and B), 1: standpipe; 2: screw; 3:nut; 4: transition area from thin to fat; 5: porous partition that prevents the scaffold from moving up and down, and allows water to pass through; 6: pin to prevent water from flowing away from the empty space in the center of the scaffold; 7: 3D printed scaffold; 8: experiment area; 9: porous partition that supports the scaffold and the pin, and allows water to pass through; 10: water outlet; 11: overflow trough; 12: stopwatch. For the radial permeability setup (D and E), 1: standpipe; 2: water pipe used to obtain the water pressure at the inner scaffold; 3: 3D printed scaffold; 4: spacer for height adjustment; 5: experiment area; 6: water pipe used to obtain the water pressure at the outer scaffold; 7: water outlet; 8: beaker; 9: stopwatch; 10: water inlet; 11: screw; 12: nut. Scale bars, 10 mm (C and F).

**Table S1.** Design parameters of five types of scaffold models.

| Scaffold groups | Filament distances (μm) | *NCA* | Branching angles α (°) |
| --- | --- | --- | --- |
| 0°/90° | 730 | - | - |
| Iteration 0 | - | 82 | - |
| Iteration 1 | - | 58 | 8 |
| Iteration 2 | - | 35 | 17; 10 |
| Iteration 3 | - | 21 | 33; 25; 12 |

**Table S2.** Design parameters of 3D bio-printing of the 3-iterations fractal-like scaffolds.

| Scaffold groups | *NCA* | Branching angles α (°) | Gradient  (From inner to outer) |
| --- | --- | --- | --- |
| Iteration 3 | 12 | 41; 33; 18 | Decrease |

**Table S3.** Design parameters of three types of scaffold models with different gradients.

| Scaffold groups | Filament distances (μm) | *NCA* | Branching angles α (°) | Gradient  (From inner to outer) |
| --- | --- | --- | --- | --- |
| 0°/90° | 986 | - | - | Constant |
| Iteration 0 | - | 50 | - | Increase |
| Iteration 3 | - | 12 | 40; 30; 12.8 | Decrease |

**Table S4.** Comparison of various design methods for bone tissue engineering scaffolds with porous graded structures.

| Design method | Printing technique | Printing complexity | Axial gradient | Radial gradient | Mixed gradient | References |
| --- | --- | --- | --- | --- | --- | --- |
| Triply periodic minimal surfaces | Powder bed fusion AND Light curing molding | High | ✔ | ✔ | ✔ | [[6-9](#_ENREF_6)] |
| Topology optimization | Powder bed fusion AND Light curing molding | High | ✔ | ✔ | ✔ | [[10-12](#_ENREF_10)] |
| Voronoi tessellation | Powder bed fusion AND Light curing molding | High | ✔ | ✔ | ✔ | [[5](#_ENREF_5), [13-15](#_ENREF_13)] |
| Lay-down pattern with different angles | Extrusion-based 3D printing | Low | ✔ | ✘ | ✘ | [[16-26](#_ENREF_16)] |
| **This work** | **Extrusion-based 3D printing** | **Low** | **✔** | **✔** | **✔** |  |

Powder bed fusion technique includes electron beam melting (EBM), selective laser melting (SLM), selective laser sintering (SLS) and so on. Light curing molding technique includes stereolithography (SLA), digital light processing (DLP) and so on. Extrusion-based 3D printing technique includes direct ink writing (DIW), fused deposition modeling (FDM), robocasting and so on.

**Movie S1.** Design of bone-mimicking scaffolds based on fractal design. The movie demonstrates the design of fractal-like bone scaffolds with 0-iterations and 3-iterations fractal-like structures. The fractal-like scaffolds with different gradients can be obtained by adjusting the number of iterations, the filament diameter (*D*_Filament_), the number of arrays (*NCA*), and the branching angles (*α*).

**Movie S2.** EB-3D printing of conventional scaffolds with a lay-down pattern of 0°/90°. The movie play speed is 5 times (5X) fast-forward. The movie shows the 3D printing of a two-layer structure of traditional scaffolds with lay-down patterns of 0°/90°.

**Movie S3.** EB-3D printing of fractal-like scaffolds with 0 iterations. The movie play speed is 5 times (5X) fast-forward. The movie shows the 3D printing of a two-layer structure of fractal-like scaffolds with 3 iterations. The two-layer structure can be used as a cell, and the cell is arrayed along the Z-axis according to the height of the required scaffold. The fractal layer and the ring layer support each other.

**Movie S4.** EB-3D printing of fractal-like scaffolds with 3 iterations. The movie play speed is 5 times (5X) fast-forward. The movie shows the 3D printing of a two-layer structure of fractal-like scaffolds with 3 iterations. The two-layer structure can be used as a cell, and the cell is arrayed along the Z-axis according to the height of the required scaffold. The fractal layer and the ring layer support each other.

**Movie S5.** EB-3D bio-printing of fractal-like scaffolds with 3 iterations. The movie play speed is 5 times (5X) fast-forward. The movie shows the 3D bio-printing of novel fractal-like scaffolds with 3 iterations. Milky white and green materials are β-TCP/PCL and dye/Alg inks, respectively. The two-layer structure with the gap filled the bio-ink can be used as a cell, and the cells are arrayed along the Z-axis according to the height of the required scaffold. The filaments of adjacent layers support each other.
